# Detecting Individual Sites Subject to Episodic Diversifying Selection

**DOI:** 10.1371/journal.pgen.1002764

**Published:** 2012-07-12

**Authors:** Ben Murrell, Joel O. Wertheim, Sasha Moola, Thomas Weighill, Konrad Scheffler, Sergei L. Kosakovsky Pond

**Affiliations:** 1 Biomedical Informatics Research Division, eHealth Research and Innovation Platform, Medical Research Council, Tygerberg, South Africa; 2 Computer Science Division, Department of Mathematical Sciences, Stellenbosch University, Stellenbosch, South Africa; 3 Department of Pathology, University of California San Diego, La Jolla, California, United States of America; 4 Department of Medicine, University of California San Diego, La Jolla, California, United States of America; Fred Hutchinson Cancer Research Center, United States of America

## Abstract

The imprint of natural selection on protein coding genes is often difficult to identify because selection is frequently transient or episodic, i.e. it affects only a subset of lineages. Existing computational techniques, which are designed to identify sites subject to pervasive selection, may fail to recognize sites where selection is episodic: a large proportion of positively selected sites. We present a mixed effects model of evolution (MEME) that is capable of identifying instances of both episodic and pervasive positive selection at the level of an individual site. Using empirical and simulated data, we demonstrate the superior performance of MEME over older models under a broad range of scenarios. We find that episodic selection is widespread and conclude that the number of sites experiencing positive selection may have been vastly underestimated.

## Author Summary

Identifying regions of protein coding genes that have undergone adaptive evolution is important to answering many questions in evolutionary biology and genetics. In order to tease out genetic evidence for natural selection, genes from a diverse array of taxa must be analyzed, only a subset of which may have undergone adaptive evolution; the same gene region may be under stabilizing or relaxed selection in lineages leading to other taxa. Most current computational methods designed to detect the imprint of natural selection at a site in a protein coding gene assume the strength and direction of natural selection is constant across all lineages. Here, we present a method to detect adaptive evolution, even when the selective forces are not constant across taxa. Using a variety of well-characterized genes, we find evidence suggesting that natural selection is generally episodic and that modeling it as such reveals that many more sites are subject to episodic positive selection than previously appreciated.

## Introduction

Following the introduction of computationally tractable codon-substitution models [1], [2] nearly two decades ago, there has been sustained interest in using these models to study the past action of natural selection on protein coding genes. Positive selection can be inferred whenever the estimated ratio (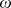) of non-synonymous (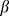) to synonymous (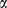) substitution rates significantly exceeds one (reviewed in [3] and [4]). In the original models, the 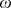 ratio was shared by all sites in an alignment, providing little power to detect the signature of positive selection. Indeed, even among classical examples of positively selected genes [5], [6], [7], most substitutions are expected to be neutral or deleterious [8]. Consequently, relatively few genes in which mean 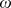 estimates are significantly greater than one are expected to exist, e.g. only 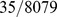 were found in a human - chimpanzee genome-wide comparison [9].

**Figure 1:**
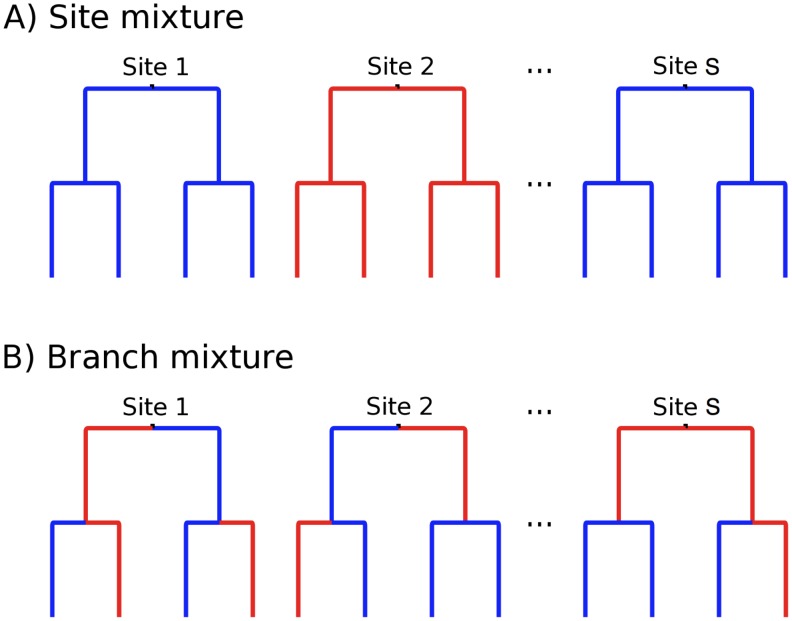
The standard random effects approach and samples. A) The standard random effects approach, in which the rates vary randomly over sites but are constant over branches. Different values of 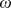 are showed in different colors. B) Samples from our new random effects approach [20], used by MEME, in which the rate on each branch is drawn independently of the rate on any other branch. All possible assignments of rates to sites are considered.

Random effects codon-substitution models [10] permitted 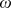 to vary from site to site, which made it possible to identify instances when positive selection had acted only upon a small proportion of sites. Such site-level models can detect which positions in a sequence alignment may have been influenced by diversifying positive selection, e.g. [11], [12]. However, these models posit that diversifying selective pressure at each site remains constant throughout time, i.e. affects most lineages in the phylogenetic tree, (Figure 1A), and there are very few cases where this assumption is biologically justified (see [13], [14], [15], [16] for examples of models that allow selection to vary throughout the tree). When a site evolves under purifying selection on most lineages, site methods which assume 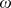 is constant over time may be unable to identify any episodic positive selection, since they will likely infer 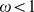 [17]. It has been noted that positive selection is more readily identified in smaller alignments: counterintuitively, including additional sequences may cause sites to no longer be detected [18], [19]. This phenomenon could be readily explained by purifying selection on some lineages masking the signal of positive selection on others.

We present a mixed effects model of evolution (MEME), based on the broad class of branch-site random effects phylogenetic methods recently developed by our group [20]. MEME allows the distribution of 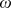 to vary from site to site (the fixed effect) and also from branch to branch at a site (the random effect, Figure 1B). Our approach provides a qualitative methodological advance over existing approaches which integrate site-to-site and lineage-to-lineage rate variation, e.g. the branch-site methods [17] or codon-based covarion models [13]. MEME can reliably capture the molecular footprints of both episodic and pervasive positive selection, a task for which current models are not well suited. Using empirical sequence data sets spanning diverse taxonomic categories and gene functions, along with comprehensive simulations, we demonstrate that MEME matches the performance of traditional site methods when natural selection is pervasive, and that MEME reliably identifies episodes of diversifying evolution affecting a small subset of branches at individual sites, where site methods often report purifying selection at the same site. For most empirical data sets analyzed here, episodic selection appears to be the dominant form of adaptive evolution. The biological implications of this type of selection are discussed for each specific data set. We conclude by providing practical guidelines for applying MEME to biological data, and argue that while it is possible to reliably identify sites or branches subject to episodic diversifying selection, statistical power to detect individual branch-site pairs evolving adaptively is inherently limited by a small sample size available for such inference.

## Methods

At its core, our approach uses phylogenetic models to describe the evolution of codon characters along a branch in a phylogeny by a continuous-time stationary Markov process. Given a phylogenetic tree 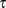, with 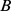 branches and a vector of relative branch length parameters 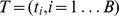, the probability of changing from codon 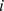 to 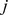 at a site along branch 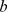 in time 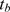, is recorded in the 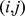 element of the transition matrix 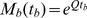, where 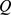 is the rate matrix. The elements 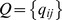 parameterize the instantaneous rate of substitution of codon 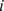 with codon 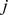:
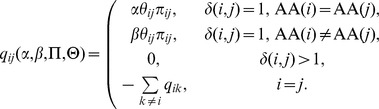


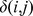
 counts the number of nucleotide differences between codons 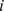 and 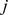. 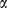 and 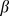 parameterize the rates of synonymous and non-synonymous substitutions, respectively. 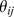 (comprising 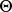) are the nucleotide mutational biases, which we model using the 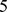-parameter general time reversible nucleotide model. 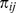 (comprising 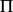) denote the equilibrium frequency parameters. Our estimate (denoted throughout as 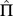) uses nine position-specific frequency parameters for the target nucleotides [1], corrected for the absence of stop codons using the 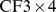 estimator [21]. The likelihood of observing the site is calculated using the pruning algorithm [22] given the data, the tree (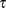), the instantaneous rate matrix (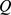), and the branch lengths (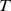).

To model the evolution of a site in an alignment in a manner that treats the non-synonymous rate (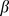) at each branch 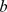 as a random draw from one of 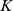 selective categories, we introduce a variable, 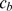, which can take values from 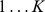. An assignment of categories to all 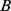 branches, is described by the configuration vector 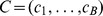 of branch categories. We assume that the category on each branch is independent of that on all other branches, and that each category has an associated probability, 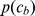, for each branch. Next, we seek to marginalize the likelihood of each site 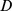 over all branch configuration vectors:
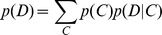
Since this sum is over possible configurations, it has 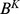 terms, and would appear infeasible, unless 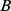 is small. However, if we assume that branch categories are independent, 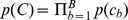, then the sum can be computed directly using the pruning algorithm by replacing the transition matrices with mixtures of transition matrices (see [20] for the derivation). If 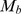 is the transition matrix on branch 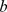, and we denote Felsenstein's algorithm, which computes the probability of observing 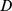 given a transition probability matrix for every branch, as 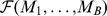, then:
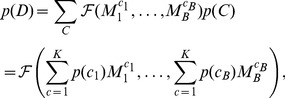
(1)where 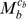 associates a transition matrix at each branch with a category. We have thus constructed a tractable model where the process at every branch is a random draw from a set of 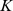 categories.

In [20], we used this result to develop a model where each branch had a set of 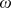 values and proportion parameters common to all sites. The goal was to identify lineages with a proportion of sites evolving with 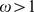. Here, we let each site have a set of free parameters governing the strength of selection for two discrete categories, and weights for each category, and these parameters are shared for all branches at that site. The goal is to detect sites where a proportion of lineages are evolving with 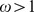.

### The MEME test for episodic diversifying selection

The fitting of MEME to an alignment of coding sequences proceeds in three stages:

First, the 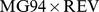 codon model with an alignment-wide 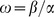 is fitted to the data using parameter estimates under a GTR nucleotide model as initial values. Although in some cases nucleotide branch lengths may be a good approximation to codon branch lengths [23], [24], recent results indicate that in other instances, nucleotide models can significantly underestimate branch lengths and possibly bias downstream inference [25]. The resulting maximum likelihood estimates, 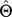 and 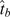, for each branch 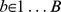, are used in the site-by-site analyses in the next two steps. Thus we are assuming that the relative branch length and mutational bias parameters are shared across sites and are well approximated by those estimated under a simpler codon model. However, the absolute branch lengths also depend on the site- and model-specific rate parameters below.

Second, at each site, we first fit the alternative random effects model of lineage-specific selective pressure with two categories of 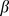: 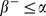 and 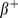 (unrestricted). The probability (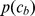 in equation 1) that branch 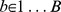 is evolving with 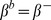, is 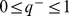, and the complementary probability that it is evolving with 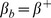 is 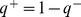. By equation 1, the phylogenetic likelihood at a site, marginalized over all 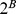 possible joint assignments of 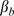, is equivalent to computing the standard likelihood function with the following mixture transition matrix for each branch 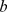:
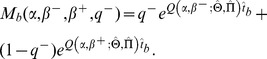
(2)


Consequently, the alternative substitution model includes four parameters for each site, inferred jointly from all branches of the tree: 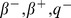 and 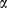. These form the fixed effects component of the model. Estimating 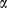 separately for each site accounts for the site-to-site variability in synonymous substitution rates [26].

Lastly, at every site, we fit the model from the previous step, but with 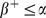: our null model. Using simulated data, we determined that an appropriate asymptotic test statistic for testing most worst-case null of of 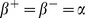 is a 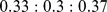 mixture of 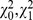 and 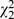 (see Text S1). Mixture statistics of this form often arise in hypothesis testing where model parameters take values on the boundaries of the parameter space, and closed-form expressions for mixing coefficients are difficult to obtain [27].

Throughout the manuscript, we compare MEME to the fixed effects likelihood approach, introduced in [24] (see Text S1 for motivation). The procedure used by FEL differs from MEME in that a single pair of 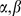 rates are fitted at each site (no variation over branches) in Step 2, and the test in Step 3 is to determine if 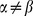. Positive selection is inferred by FEL when 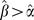 and the p-value derived from the LRT is significant, based on the 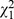 asymptotic distribution.

### Detecting individual branches subject to diversifying selection at a given site

If the LRT indicates that a particular site (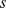) is subject to episodic diversifying selection, it may be of interest to explore which branches at that site have undergone diversification. The empirical Bayes (EB) procedure originally used to identify individual sites subject to diversifying selection in random effects models [28], can be readily adapted here. To compute the empirical posterior probability at branch 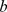 that 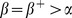, we apply Bayes' theorem, using 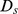 to denote the data at site 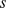 and 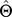 to denote all the maximum likelihood parameter estimates from the alternative MEME model fitted to site 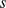:
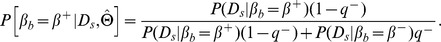
To compute the two likelihood terms 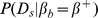 and 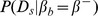, we use 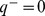 and 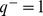, respectively, for the model assigned to branch 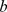 in equation 2. The rest of the branches employ the matrices fitted under the alternative model of MEME. Having computed 
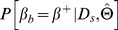
 for each branch 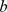, we evaluate the empirical Bayes factor for the event of observing positive selection at each branch:
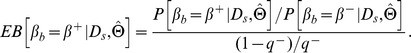
When 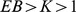, sequence data increase the prior odds of observing selection at the branch. We do not recommend using this type of inference other than for the purposes of data exploration, even for large values of 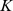 (e.g. 100). Intuitively, all the information contributing to the estimate of 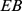 is derived from observing the evolution along a single branch at a single site (i.e. from a sample with size 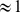). To quantify this supposition, we simulated sequence data using the vertebrate rhodopsin phylogeny and branch lengths, applied positive selection of varying strength to five branches in the tree selected *a priori* (see Text S1), and applied the EB procedure to infer the identity of selected branches.

## Results

### Model assessment

To assess the performance of MEME on both simulated and empirical data, we selected the fixed effects likelihood method (FEL [24]) as the most appropriate reference test for pervasive diversifying selection, because FEL most closely matches the assumptions made by MEME (see Text S1). We simulated data sets under a number of scenarios: refer to Text S1 for details of simulation strategies.

#### Assessing the rates of false positives

Under the scenario where each site was evolved under the worst-case null hypothesis of constant 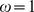, MEME had well controlled rates of false positives at test p-value of 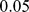 (Figure S1, also see Text S1 for the empirical derivation of the asymptotic distribution of the test statistic for this hypothesis). MEME appears to be conservative for smaller sample sizes (numbers of sequences, 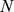), but not for larger samples. The rates of false positives were 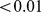 (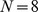), 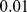 (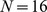), 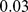 (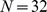), 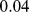 (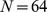), and 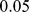 (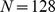 and 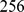). We also analyzed simulations based on seven large (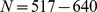) phylogenies downloaded from TreeBase (http://www.treebase.org). The rate of false positives remained well controlled (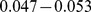) at a nominal p-value of 0.05, suggesting that further increasing the number of taxa does not lead to a degradation of Type I error rates.

A further analysis using 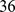 trees from a variety of published studies downloaded from TreeBase, to simulate 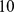 replicates from each tree (see Text S1 and Tables S1 and S2 for details), revealed that MEME is generally conservative for alignments of with low pairwise divergence (e.g. 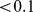 nucleotide substitutions per site), nominal for those with medium to high pairwise divergence (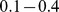 nucleotide substitutions per site), and nominal to slightly anti-conservative for higher pairwise divergence (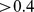 nucleotide substitutions per site), although this relationship is influenced by other factors. Overall, we conclude that false positive rates of MEME, are well controlled in the setting of the most pessimistic (strict neutral) null.

#### Constant selection pressure at individual sites

At nominal 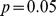 MEME consistently tracked FEL on sequence alignments simulated under the lineage-constant model assumed by FEL (Table S3), losing several percentage points of power because of its more conservative test statistic. Because each simulated alignment contained a subset of sites generated under the null (neutral model), we could derive empirical estimates of the size of the test and set the nominal p-value to achieve a Type I error rate of 5%. When calibrated to deliver a 5% Type I error rate, MEME held a small edge in power. This finding is not surprising, because at a fixed Type I rate, MEME should find every site found by FEL, and resolve FEL borderline cases affected by stochastic variation in 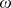 throughout the tree.

#### Variable selection pressure at individual sites

The difference in power between MEME and FEL became stark when selection at individual sites varied among lineages, with each branch evolving under positive selection (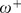) with probability 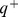, and negative selection (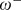) with complimentary probability 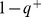. For every combination of independent simulation parameters (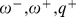), MEME had more power to detect sites under episodic diversifying selection (Table 1). Both methods gained power with an increasing proportion of positively selected lineages and/or a greater degree of diversification. The largest differences between MEME and FEL were observed when a small proportion of lineages (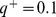) were subjected to diversifying selection. Regardless of the strength of background purifying selection, FEL was effectively powerless (power 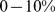) to detect episodes of positive selection under any of the three phylogenetic simulation scenarios, whereas MEME achieved low (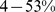 when 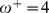), modest (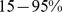 when 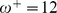), and excellent (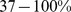 when 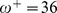) power. Under these conditions, the power of MEME increased with the alignment size, whereas the power of FEL remained very low. Although FEL gained appreciable power when 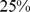 (or 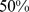) of the lineages were subject to diversification, its power was on average only 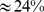 (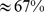) of that realized by MEME.

**Table 1:** Comparative performance of FEL and MEME on simulated data where 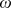 varies along phylogenetic lineages.

		Japanese encephalitis virus *env*			Vertebrate rhodopsin			Camelid VHH		
ω^**−**^	*q* ^**+**^	ω^**+**^ = 4	ω^**+**^ = 12	ω^**+**^ = 36	ω^**+**^ = 4	ω^**+**^ = 12	ω^**+**^ = 36	ω^**+**^ = 4	ω^**+**^ = 12	ω^**+**^ = 36
0	0.1	0.00 **0.06**	0.01 **0.25**	0.03 **0.50**	0.00 **0.21**	0.00 **0.53**	0.02 **0.81**	0.00 **0.53**	0.00 **0.95**	0.04 **0.99**
0	0.25	0.01 **0.12**	0.06 **0.32**	0.12 **0.51**	0.01 **0.30**	0.04 **0.68**	0.15 **0.88**	0.00 **0.66**	0.14 **0.98**	0.56 **1.00**
0	0.5	0.06 **0.12**	0.19 **0.29**	0.34 **0.45**	0.09 **0.28**	0.34 **0.59**	0.54 **0.82**	0.23 **0.77**	0.85 **0.98**	0.96 **0.98**
0.2	0.1	0.00 **0.05**	0.01 **0.21**	0.02 **0.41**	0.00 **0.09**	0.01 **0.35**	0.02 **0.67**	0.00 **0.16**	0.01 **0.87**	0.04 **0.98**
0.2	0.25	0.02 **0.08**	0.07 **0.27**	0.14 **0.48**	0.03 **0.17**	0.09 **0.55**	0.17 **0.84**	0.01 **0.42**	0.27 **0.96**	0.62 **0.99**
0.2	0.5	0.05 **0.11**	0.18 **0.29**	0.36 **0.49**	0.13 **0.25**	0.36 **0.60**	0.55 **0.76**	0.30 **0.72**	0.84 **0.99**	0.90 **0.99**
0.4	0.1	0.00 **0.04**	0.01 **0.15**	0.03 **0.37**	0.01 **0.07**	0.02 **0.30**	0.03 **0.57**	0.01 **0.10**	0.04 **0.78**	0.10 **0.97**
0.4	0.25	0.02 **0.06**	0.09 **0.27**	0.15 **0.45**	0.04 **0.16**	0.09 **0.49**	0.21 **0.78**	0.03 **0.32**	0.33 **0.97**	0.63 **0.99**
0.4	0.5	0.07 **0.10**	0.17 **0.26**	0.33 **0.46**	0.17 **0.28**	0.39 **0.58**	0.51 **0.76**	0.40 **0.62**	0.82 **0.94**	0.96 **1.00**

Power to detect sites under selection (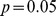) are reported for FEL and MEME (in **boldface**) for each unique combination of negative selection (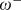), positive selection (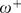), and proportion of branches under positive selection (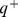) parameters.

Taken together, the constant and variable selection pressure simulations demonstrate the uniform superiority of MEME over a standard test for diversifying positive selection. MEME has well controlled rates of false positives, has power comparable to FEL when selective forces are uniform at individual sites, and gains a large power advantage when these forces are variable, as is undoubtedly the case in most biological data sets.

#### Power and accuracy of the empirical Bayes procedure to identify branches subject to diversifying selection at a single site

Our exploratory simulations (see Figure S2) suggest that it is difficult to accurately identify individual positively selected branches at an individual site. We restricted the analysis to only those sites, which were found to be under episodic diversifying selection by MEME (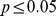) and set the threshold of 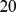 for the empirical Bayes factor to call an individual branch selected. The best results are achieved when selected branches are placed in the background of strongly conserved lineages (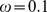) – an individual branch is correctly detected in approximately 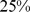 of cases, while *at least* one selected branch is found in 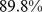 of cases (see Figure S3). However, while none of the negatively selected background branches are reported in more than 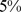 of cases, in 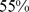 of cases *at least* one background branch was falsely detected as positively selected. In a more difficult case of neutrally evolving background, the EB procedure performs considerably worse: at least one select branch is found in 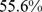 of cases, whereas at least one background branch is detected in 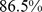 instances. 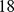 background neutral branches are reported as selected at over 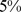 frequency, while the 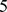 positively selected branches are identified at 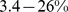 of selected sites.

### Empirical data

To gauge the comparative performance of MEME and FEL when identifying sites subject to pervasive diversifying selection, we used a collection of 16 protein-coding alignments, representing a diverse array of taxa, genes subject to differing levels of conservation, and a range of data set sizes (Table 2). In 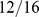 alignments analyzed, MEME identified all the sites inferred by FEL to be under diversifying positive selection and found between 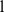 (e.g. West Nile virus NS3) and 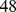 (Diatom SIT) additional sites that were subject to episodic diversifying selection (Table 2). In four data sets, 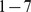 sites identified by FEL with p-values close to 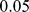 were missed by MEME. Note that MEME p-values for these sites remained in the 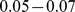 range (Table 2), i.e. marginally significant.

**Table 2:** Comparative performance of MEME and FEL on 16 empirical alignments (see Results and Text S1 for an extended discussion of each individual case).

Data set	N	S	Mean	Classes of sites detected at *p*≤0.05				Mean *q* ^**+**^		Sites where
			Div.	M^+^F^0^	M^+^F^+^	M^+^F^−^	M^−^F^+^	M^+^F^0−^	M^+^F^+^	MEME>FEL at *p* = 0.05
Abalone sperm lysin	25	134	0.43	17	9	0	1 (0.04/0.05)	0.17	0.35	19
Camelid VHH	212	96	0.27	22	6	2	0 (n/a)	0.11	0.50	26
Diatom SIT	97	300	0.54	12	0	36	0 (n/a)	0.05	n/a	82
Drosophila *adh*	23	254	0.26	9	1	0	0 (n/a)	0.09	0.19	7
Echinoderm H3	37	111	0.33	0	0	1	0 (n/a)	0.02	n/a	3
Flavivirus NS5	18	342	0.48	3	0	1	0 (n/a)	0.16	n/a	7
Hepatitis D virus Ag	33	196	0.29	13	7	0	1 (0.05/0.07)	0.08	0.37	10
HIV-1 *rt*	476	335	0.08	12	10	7	0 (n/a)	0.04	0.69	27
HIV-1 *vif*	29	192	0.08	5	2	0	7 (0.04/0.06)	0.11	0.59	3
IAV H3N2 HA	349	329	0.04	7	11	2	3 (0.04/0.06)	0.04	0.73	8
JEV *env*	23	500	0.13	2	1	1	0 (n/a)	0.11	1.00	3
Mamallian 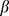 -globin	17	144	0.38	10	2	0	0 (n/a)	0.20	0.31	11
Primate *COXI*	21	510	0.36	3	0	1	0 (n/a)	0.18	n/a	4
Salmonella *recA*	42	353	0.04	1	0	0	0 (n/a)	0.02	n/a	0
Vertebrate rhodopsin	38	330	0.34	13	1	5	0 (n/a)	0.11	0.74	39
West Nile virus NS3	19	619	0.13	1	1	0	0 (n/a)	0.04	1.00	2
Total/Mean				130	51	56	12	0.10	0.59	

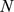
 (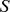) reports the number of sequences (codons) in the alignment. 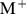 (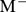) refers sites found by MEME to be positively (negatively) selected (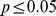). 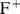 (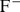) denote sites found by FEL to be positively (negatively) selected (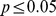). 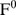 references sites that are classified as neutrally evolving by FEL. Values in parentheses for the 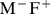 column show the mean p-values for FEL and MEME on this set of sites, respectively. Values reported in the rightmost column count the number of sites where MEME fits significantly better than FEL, based on a 2-degrees of freedom LRT (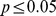). Abbreviations: IAV = Influenza A virus, JEV = Japanese encephalitis virus.

Sites identified by both methods tended to have a greater average proportion of lineages under selection (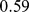, measured by the mean of MLE estimates of 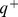); sites found only by MEME experienced more episodic selection (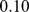). In 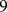 data sets (Table 2), sites that FEL inferred to be under purifying selection are instead identified by MEME as likely to have been subjected to episodic diversifying selection. Almost universally (Tables S4, S5, S6, S7, S8, S9, S10, S11, S12, S13, S14, S15, S16, S17, S18, S19), such sites had a smaller estimated proportion of positively selected lineages (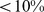). This behavior is consistent with the relative performance of the two tests on simulated data and corroborates the expectation that MEME has greater power to identify sites when only a proportion of lineages evolved under positive selection. Vertebrate rhodopsin, Japanese encephalitis virus *env*, and Camelid VHH are investigated in detail below; for a discussion other genes, see Text S1.

### Vertebrate rhodopsin

The vertebrate rhodopsin (a low-light vision protein) data set was previously experimentally investigated for the substitutions that modulate the wavelength of the light absorbed by the molecule (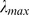, [18]). The authors asserted that, because none of the 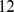 sites that they had determined as affecting 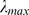 by site-directed mutagenesis were detected by site-level computational methods, “statistical tests of positive selection can be misleading without experimental support.” Other authors reanalyzed the same data set more comprehensively and went even further, questioning the utility of 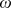-based methods for detecting experimentally validated sites, because “most of the current statistical methods are designed to identify codon sites with high 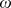 values, which may not have anything to do with functional changes. The codon sites showing functional changes generally do not show a high 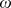 value” [29]. The validity of this generalization has been correctly questioned with a simple counter-argument that the sites detected by computational methods may also be functionally important, because the change in 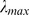 is unlikely to be the sole determinant of adaptation [17].

The MEME analysis of this gene suggests another obvious alternative, also expounded by previous studies [17]: the failure of the original computational analysis [18] to identify functionally important sites results from the fact that these sites have been subjected to episodic selection, which is masked by predominantly purifying selection elsewhere in the tree. Indeed, among three sites that alter 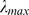 found by MEME (96, 183 and 195, versus none found by FEL), no more than  of the branches exhibited 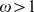 (Table S17); at these sites, the average 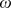 is less than 1. We note that, because adaptive evolution will not always adhere to a single, simple scenario of episodic diversifying selection, we do not expect MEME to find all 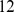 sites experimentally confirmed to alter 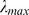. For example, three of the nine missed sites (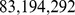) appear to have been subjected to partial selective sweeps and have been detected using a specialized model of directional evolution [29].

**Figure 2:**
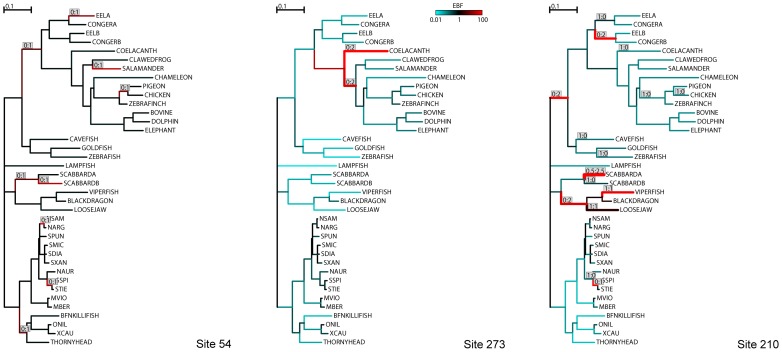
Individual sites of the vertebrate rhodopsin alignment used to illustrate similarities and differences between FEL and MEME. Branches that have experienced substitutions, based on most likely joint maximum likelihood ancestral reconstructions at a given site, are labeled as count of synonymous substitutions:count of non-synonymous substitutions. The thickness of each branch is proportional to the minimal number of single nucleotide substitutions mapped to the branch. Branches are colored according to the magnitude of the empirical Bayes factor (EBF) for the event of positive selection: red – evidence for positive selection, teal – evidence for neutral evolution or negative selection, black –Ê no information. See Methods for more detail. All three sites were identified as experiencing positive diversifying selection by MEME. FEL reported site 54 as positively selected, site 273 as neutral, and site 210 as negatively selected.

Three sites from this alignment can be used to illustrate how the inclusion of lineage variability modifies inference of selection (Figure 2). Site 54 was inferred to have experienced pervasive non-synonymous substitutions throughout its evolutionary history. Both FEL and MEME detect this site as positively selected (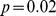). Sixty three percent of the lineages at this site evolved with 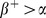, whereas the remainder were conserved (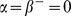), according to MEME. The log-likelihood of the site is only marginally higher for MEME, which suggests that MEME behaves like FEL at sites with “canonical” patterns of diversifying selection, corroborating the simulation results.

At codon 273, FEL obtained a maximum likelihood estimate of 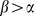, but failed to infer positive selection, as the signal was not statistically significant (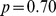). MEME, on the other hand, allocated 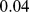 (0.013–0.10: 95% confidence interval obtained by latin hypercube sampling importance resampling [30]) of branches to a rate class with 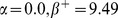 (2.94–6726) and inferred positive selection (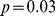). The difference in log-likelihoods between MEME and FEL is 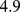 points: MEME fits significantly better, based on a 2-degrees of freedom likelihood ratio test (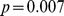). The maximum likelihood estimates of individual model parameters have large associated errors (although in all posterior samples we obtained 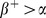), as is expected for inference based on a single site. This has also been noted by Yang and dos Reis [17]. The point estimates themselves, however, are immaterial for inferring whether or not a site is positively selected, since the likelihood ratio test is used for that purpose.

Perhaps the most dramatic example of the added power of MEME is illustrated by site 210. At this site, the evolutionary history is replete with non-synonymous substitutions along deep lineages followed by extensive synonymous evolution, indicative of purifying selection. There is also a small clade with repeated synonymous and nonsynonymous substitutions. Averaging over all branches, FEL determined that the site, overall, is under negative selection (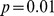). MEME reported that 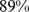 of the branches were under a very strong selective constraint (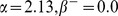), but that the remaining 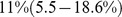 were under strong diversifying selection (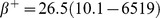). The log-likelihood improvement is now 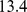 at the cost of two parameters, which is highly significant (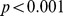). Site 210 is the ideal illustration of why it is undesirable to average 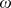 over all lineages: bursts of diversification followed by conservation will most likely be missed by traditional site methods.

### Japanese encephalitis virus *env*

No evidence for selection was found in this envelope gene in previous analyses [28], and FEL found only one site under positive selection. Despite the low levels of divergence among a relatively small number of taxa (23 isolates), MEME found episodic selection at sites called negatively selected by FEL (Table S12). Two of these sites fall within a beta-barrel epitope known to be involved in escape from neutralizing antibodies [31]. Sites 33 and 242 showed evidence of repeated toggling at terminal lineages. Remarkably, site 33 – likely a part of a neutralizing antibody epitope [32] – changed from isoleucine to leucine on 6 terminal lineages; site 242 changed from phenylalanine to serine on 5 terminal lineages. These substitutions co-occur on three terminal lineages. Evidence of recombination was detected in this alignment, and corrected for using a partitioning approach (details on how MEME can correct for recombination are in Text S1).

### Camelid VHH

The camelid VHH data set comprises partial variable domain sequences (germline alleles) of llama and dromedary heavy chain only antibodies (Table S3). 11 of 16 sites in the variable complementarity determining regions (CDR) 1 (sites 26–33) and 2 (sites 51–58) were found to be under diversifying selection by MEME (2/16 were detected by FEL and 2 more were marginally significant). Because CDR regions are driven to diversify in order to provide a broad basis of antigen recognition, positive selection is expected to be commonplace in the CDRs [33]. MEME was able to uncover selective signatures at a majority of those sites. Of the remaining 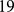 sites classified by MEME as positively selected, six were associated with VHH family differentiation [34]. Unlike standard antibodies, which must maintain relatively conserved framework regions (FR) involved in binding heavy and light chains to form functional tetramers, VHH antibodies are free of such functional constraints. A previous analysis of camelid VHH for evidence of positive selection using counting methods [35] reported evidence for positive selection at a single site (14) in FR1 (sites 1–25 in Table S3), but this analysis could find no clear evidence of positive or negative selection at 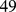 FR sites. In contrast, MEME inferred episodic selection at six sites in FR1, six sites in FR2 (sites 34–50), and 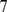 sites in FR3 (sites 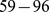). The well-known lack of power of counting methods to detect even pervasive selection [17] likely hampered the previous study.

### Effect of sequence sampling

Although a previous analysis of 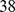 vertebrate rhodopsin sequences found no sites under selection at posterior probability 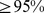 [18], the same authors found 7 selected sites in the subset of 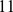 squirrelfish sequences, and 2 selected sites when the subset of 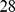 fish sequences was analyzed. These results run counter to the expectation that more data should provide greater power to detect selection. MEME, on the other hand, detects more selected sites when more sequences are included. One site is identified in the squirrelfish alignment, 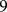 in the fish alignment, and 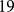 in the complete rhodopsin alignment. All but 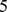 sites detected in the subset alignments are also identified in the full alignment (Table S20). Allowing 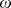 to vary over branches at least partially mitigates the pathology of constant-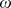 models which effectively rely on an average 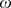 for inferring selection. A similar pattern is seen in the analysis of the influenza A virus H3N2 hemagglutinin sequences, where site-level methods also appear to be sensitive to sequence sampling ([19], see Text S1 and Table 23).

## Discussion

We have presented a mixed effects model of evolution, MEME, and a statistical test for detecting the signal of past episodic positive selection from molecular sequence data. Our model corrects the biologically unrealistic assumption that selective pressure, as measured by the 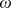 ratio, remains constant over lineages. Based on comprehensive simulations and empirical analysis of an array of taxonomically diverse genes, MEME can be recommended as a replacement for existing site models. MEME matches the performance of older approaches when natural selection is pervasive, but possesses greater power to identify sites where episodes of positive selection are confined to a small subset of branches in a phylogenetic tree.

Our results suggest that it may be necessary to revise previous estimates of the proportion of sites under positive selection in many genes. Using the FEL method, which assumes constant selective pressure at a site, we are able to detect 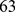 sites across all 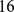 empirical alignments. MEME identifies 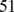 of these sites (the remaining 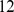 are borderline significant) and 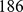 additional sites – nearly 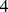 times as many as FEL. For individual data sets (e.g. Drosophila *adh* and Diatom SIT, Table 2), the differences may be even more dramatic. The greater power of MEME indicates that selection acting at individual sites is considerably more widespread than constant 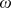 models would suggest. It also suggests that natural selection is predominantly episodic, with transient periods of adaptive evolution masked by the prevalence of purifying or neutral selection on other branches. We emphasize that MEME is not just a quantitative improvement over existing models: for 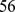 sites in our empirical analyses, we obtain qualitatively different conclusions. FEL asserts that these sites evolved under significant purifying selection, but MEME is able to identify the signature of positive selection on some branches. Furthermore, MEME is less sensitive to sampling effects that plague existing positive selection detection tools [18], [19]. Variable levels of purifying selection pressure across different lineages prevented these older methods from detecting instances of episodic positive selection; MEME is able to peer through the fog of purifying selection.

It is important to bear in mind that the mixture 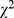 statistic used to calculate the p-values reported here is based on a null model under which all sites are evolving neutrally. This, however, is not biologically realistic: the null hypothesis against which sites ideally ought to be screened is one under which sites are evolving *either* neutrally *or* under purifying selection. But the proportion of sites evolving under negative selection and the strength of this selection are unknown and vary from case to case, which means that such a null hypothesis would be very sensitive to modeling assumptions that cannot be justified in general. Instead, the neutral null hypothesis represents a worst case scenario for our inference, so that the p-values we obtain are upper bounds of the true p-values. This ensures that our inference is conservative. Even in the worst (and biologically unrealistic) case for MEME, namely when selective pressures are constant throughout the phylogeny, the loss of power compared to FEL is minimal: a site with FEL p-values between 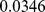 and 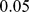 will be missed by MEME, since its p-value will be 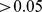 for the same ranges of the likelihood ratio test statistic (LRT). In our simulation scenarios under the assumption of constant 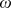, this translates to no more a 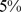 loss in power (Table S3).

Our inference is performed in a site-wise rather than an alignment-wide manner, and we therefore control the site-wise rather than the family-wise error rate. We do not recommend combining the results of multiple site-wise inferences to perform alignment-wide inference. To aid interpretation of the results while taking account of multiple testing, we calculate the false discovery rate [36]; the resulting q-value upper bounds are reported alongside their corresponding p-value upper bounds in Tables S4, S5, S6, S7, S8, S9, S10, S11, S12, S13, S14, S15, S16, S17, S18, S19. This gives an upper bound on how many of the reported sites can be expected to be false discoveries: for instance, of the 30 sites reported in Table S5 we expect no more than 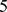 (14%) to be false, and probably far fewer because of the conservative choice of null model. We emphasize that q-values are usually much larger than their corresponding p-values and caution that p-values (regardless of whether they have been corrected for multiple testing) cannot be used to estimate an expected number of false discoveries in the same way.

MEME is a conceptual advance over the first generation of random effects models designed to detect episodic selection (called “branch-site models” in the literature [17]). MEME does not require *a priori* designation of, or an exhaustive search for, the branches under selection, and it allows each site to have its own selective history. Whereas branch-site models make restrictive *a priori* assumptions about how 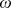 values are distributed across the tree – sometimes leading to very poor statistical performance [20] – MEME treats the selective class on each branch as a random effect that is marginalized over in the likelihood calculation.

For computational tractability, MEME assumes that the value taken by 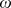 on each branch is independent of that on any other branch, i.e. selective pressures between branches are uncorrelated. This assumption could potentially be violated: for example, if 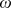 changes very slowly across the phylogeny, then 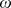 values on neighboring branches will be correlated. Further research is needed to understand how inference of selection would be affected if these correlations were directly accounted for, and whether the additional model and computational complexity would be justified. In practice, MEME could be combined with models of directional selection to improve power, e.g. [15], [16]. Unlike covarion models [37], [13], MEME does not allow 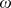 to change in the middle of a tree branch. The effect of this restriction is unclear, but it could be tested by implementing a mixed effects covarion model, where switching rates and proportion of time spent under 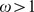 are estimated at an individual site.

The ability of MEME, or similar substitution model-based methods, to accurately infer the identity of individual branches subject to diversifying selection at a given site seems unavoidably limited. Most of the information that such inference might be based on is limited to character substitutions along a single branch at a single site, i.e. one realization of the Markov substitution process. Selection along terminal branches in the context of negatively selected background can be detected more reliably than selection along interior branches among neutrally evolving background lineages. However, we caution that despite obvious interest in identifying specific branch-site combinations subject to diversifying selection, such inference is based on very limited data (the evolution of one codon along one branch), and cannot be recommended for purposes other than data exploration and result visualization. This observation could be codified as the “selection inference uncertainty principle” – one cannot simultaneously infer both the site and the branch subject to diversifying selection. In this manuscript, we describe how to infer the location of sites, pooling information over branches; previously [20] we have outlined a complementary approach to find selected branches by pooling information over sites.

Finally, although MEME is considerably more powerful than existing methods at detecting bursts of selection, it still requires that a measurable proportion of lineages (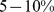) experience non-synonymous evolution at a site. When a single substitution modifies an adaptive trait and is subsequently fixed, we expect 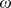 based methods to have very little power. Specialized methods which make use of change in allele frequencies [15], [16], or between and within-population diversification patterns [38], will be required in such cases.

## Supporting Information

Figure S1Quantile–Quantile plot of three asymptotic distributions (x-axis) for the MEME LRT test versus the LRT derived by parametric bootstrap (y-axis), limited to the meaningful test p-value range of 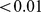. The 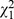 distribution is too liberal (lying below the 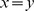 line), the 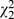 is too conservative, while the mixture is approximately correct.(PDF)

Figure S2Simulation parameters for generating datasets for evaluating the empirical Bayes inference of branch-site combinations under selection. Branches are colored according the the value of 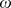 used to evolve sequences along them; branches simulated under positive selection are also labeled with 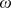 values.(PDF)

Figure S3Summary of empirical Bayes inference of branches under selection on data simulated using the selective parameters from Figure S2. Each branch is colored according to the proportion of times it was found to have an empirical Bayes factor of 20 or greater at sites with MEME p-value of 0.05 or less. Branches with 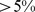 detection rates are also labeled with the values of the rates.(PDF)

Table S1False positive rates for data sets simulated under strict neutrality using empirical trees from TreeBase. The entries are sorted in order of increasing mean false positive rate derived from simulated data (10 replicates per tree). Mean divergence between any pair of leaves in a given tree is reported in expected nucleotide substitutions per site. False positive range reports the minimum and maximum values for false positive rates for an individual replicate. 95% confidence intervals are derived from the binomial distribution with the probability of success 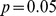, and the number of trials 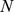 equal to the number of codons. This range provides the expected spread of per replicate false positive rates for a test that has the probability of making a false positive error of exactly 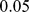 over 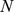 tests.(PDF)

Table S2False positive rates for three empirical trees from TreeBase when the parameters of the null model are varied: 20% of the branches are simulated with the foreground 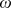, and the remainder under the background 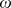. 10 replicates with 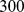 codons each per tree-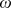 pair were simulated. The synonymous rate was set to 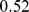 for the first 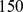 codons, 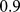 for the next 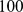 codons, and 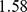 for the last 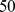 codons.(PDF)

Table S3Comparative performance of FEL and MEME on simulated data where 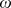 does not vary among tree branches. The rate of false positives (FP) and power are reported for a fixed nominal test p-value of 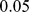. Power is also shown for the p-value that achieves FP of 0.05, estimated empirically from the distribution of p-values on the subset of sites evolving neutrally.(PDF)

Table S4Positively selected sites in abalone sperm lysin. 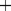 stands for a positively selected site and 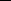 stands for a negatively selected site (FEL 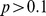). 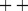 and 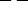 reflect borderline significant sites (FEL p between 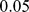 and 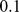). 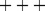 and 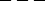 denote significant sites (FEL 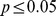).(PDF)

Table S5Positively selected sites in camelid VHH. 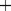 stands for a positively selected site and 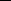 stands for a negatively selected site (FEL 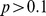). 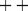 and 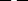 reflect borderline significant sites (FEL p between 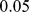 and 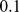). 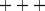 and 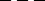 denote significant sites (FEL 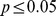).(PDF)

Table S6Positively selected sites in Diatom silicon transporters found by MEME at 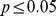. The FEL result column summarizes the classification obtained by FEL. 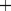 stands for a positively selected site and 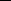 stands for a negatively selected site (FEL 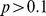). 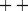 and 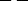 reflect borderline significant sites (FEL p between 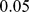 and 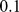). 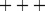 and 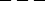 denote significant sites (FEL 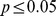).(PDF)

Table S7Positively selected sites in Drosophila *adh* found by MEME at 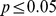. The FEL result column summarizes the classification obtained by FEL. 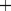 stands for a positively selected site and 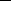 stands for a negatively selected site (FEL 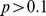). 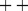 and 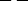 reflect borderline significant sites (FEL p between 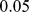 and 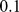). 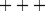 and 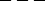 denote significant sites (FEL 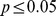).(PDF)

Table S8Positively selected sites in Echinoderm histone H3. 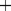 stands for a positively selected site and 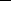 stands for a negatively selected site (FEL 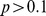). 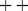 and 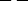 reflect borderline significant sites (FEL p between 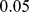 and 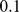). 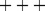 and 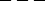 denote significant sites (FEL 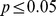).(PDF)

Table S9Positively selected sites in Flavivirus NS5. 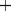 stands for a positively selected site and 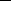 stands for a negatively selected site (FEL 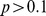). 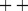 and 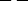 reflect borderline significant sites (FEL p between 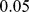 and 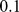). 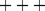 and 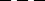 denote significant sites (FEL 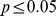).(PDF)

Table S10Positively selected sites in Hepatitis D virus Ag. 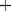 stands for a positively selected site and 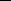 stands for a negatively selected site (FEL 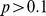). 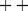 and 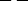 reflect borderline significant sites (FEL p between 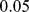 and 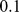). 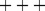 and 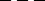 denote significant sites (FEL 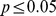).(PDF)

Table S11Positively selected sites in HIV-1 reverse transcriptase (*rt*). 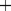 stands for a positively selected site and 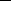 stands for a negatively selected site (FEL 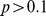). 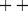 and 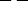 reflect borderline significant sites (FEL p between 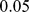 and 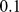). 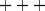 and 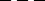 denote significant sites (FEL 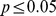).(PDF)

Table S12Positively selected sites in HIV-1 viral infectivity factor (*vif*). 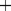 stands for a positively selected site and 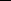 stands for a negatively selected site (FEL 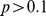). 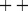 and 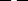 reflect borderline significant sites (FEL p between 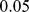 and 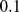). 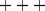 and 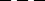 denote significant sites (FEL 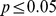).(PDF)

Table S13Positively selected sites in Influenza A virus hemagglutinin (H3N2 serotype). Superscript letters after the site indicate the epitope in which substitutions can affect phenotype. 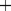 stands for a positively selected site and 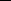 stands for a negatively selected site (FEL 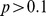). 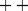 and 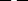 reflect borderline significant sites (FEL p between 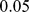 and 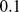). 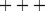 and 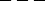 denote significant sites (FEL 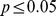).(PDF)

Table S14Positively selected sites in Japanese encephalitis virus *env*. 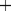 stands for a positively selected site and 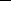 stands for a negatively selected site (FEL 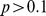). 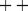 and 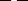 reflect borderline significant sites (FEL p between 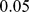 and 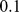). 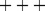 and 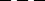 denote significant sites (FEL 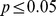).(PDF)

Table S15Positively selected sites in mammalian 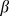-globin. The FEL result column summarizes the classification obtained by FEL. 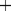 stands for a positively selected site and 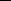 stands for a negatively selected site (FEL 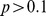). 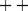 and 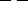 reflect borderline significant sites (FEL p between 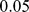 and 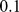). 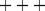 and 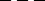 denote significant sites (FEL 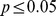).(PDF)

Table S16Positively selected sites in primate cytochrome c oxidase subunit 1 (*COX1*). 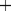 stands for a positively selected site and 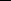 stands for a negatively selected site (FEL 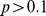). 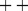 and 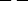 reflect borderline significant sites (FEL p between 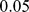 and 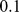). 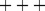 and 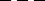 denote significant sites (FEL 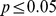).(PDF)

Table S17Positively selected sites in Salmonella *recA*. 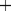 stands for a positively selected site and 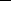 stands for a negatively selected site (FEL 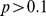). 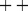 and 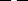 reflect borderline significant sites (FEL p between 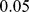 and 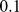). 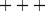 and 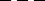 denote significant sites (FEL 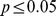).(PDF)

Table S18Positively selected sites in vertebrate rhodopsin. 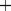 stands for a positively selected site and 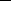 stands for a negatively selected site (FEL 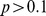). 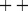 and 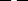 reflect borderline significant sites (FEL p between 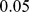 and 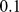). 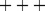 and 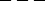 denote significant sites (FEL 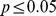).(PDF)

Table S19Positively selected sites in West Nile virus NS3. 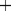 stands for a positively selected site and 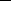 stands for a negatively selected site (FEL 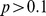). 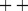 and 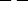 reflect borderline significant sites (FEL p between 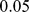 and 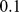). 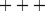 and 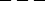 denote significant sites (FEL 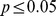).(PDF)

Table S20Test p-values for positively selected sites found by MEME in a set of 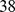 vertebrate rhodopsin sequences analyzed with REL methods in Yokoyama2008fk. Sites with 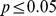 are shown in bold. The partial ordering of subsets is as follows: Squirrelfish 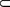 Fish 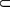 All, Coelacanth and tetrapods 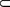 All. Sites found to be under positive selection with posterior probability of 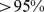 (M8 model) in Yokoyama2008fk in at least one of the subsets are marked with 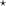.(PDF)

Table S21Test p-values for positively selected sites found by MEME in a set of 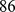 influenza A virus hemagglutinin sequences (Set 3) and its various subsets, analyzed with REL methods in Chen2011fk. Sites with 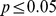 are shown in bold. The partial ordering of subsets is as follows: Set 4 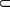 Set 1 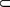 Set 3, Set 5 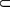 Set 2 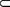 Set 3, Set 6 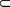 Set 3, Set 7 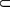 Set 3. Sites found to be under positive selection with posterior probability of 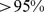 (M3 model) in Chen2011fk in at least one of the subsets are marked with 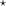.(PDF)

Text S1 Supplementary methods, results, and discussion.
(PDF)
