# Supplementary figures and images for: Detecting Individual Sites Subject to Episodic Diversifying Selection
Source: PLoS Genet. 2012 Jul 12;8(7):e1002764. doi: 10.1371/journal.pgen.1002764 (PMC3395634; doi:10.1371/journal.pgen.1002764)

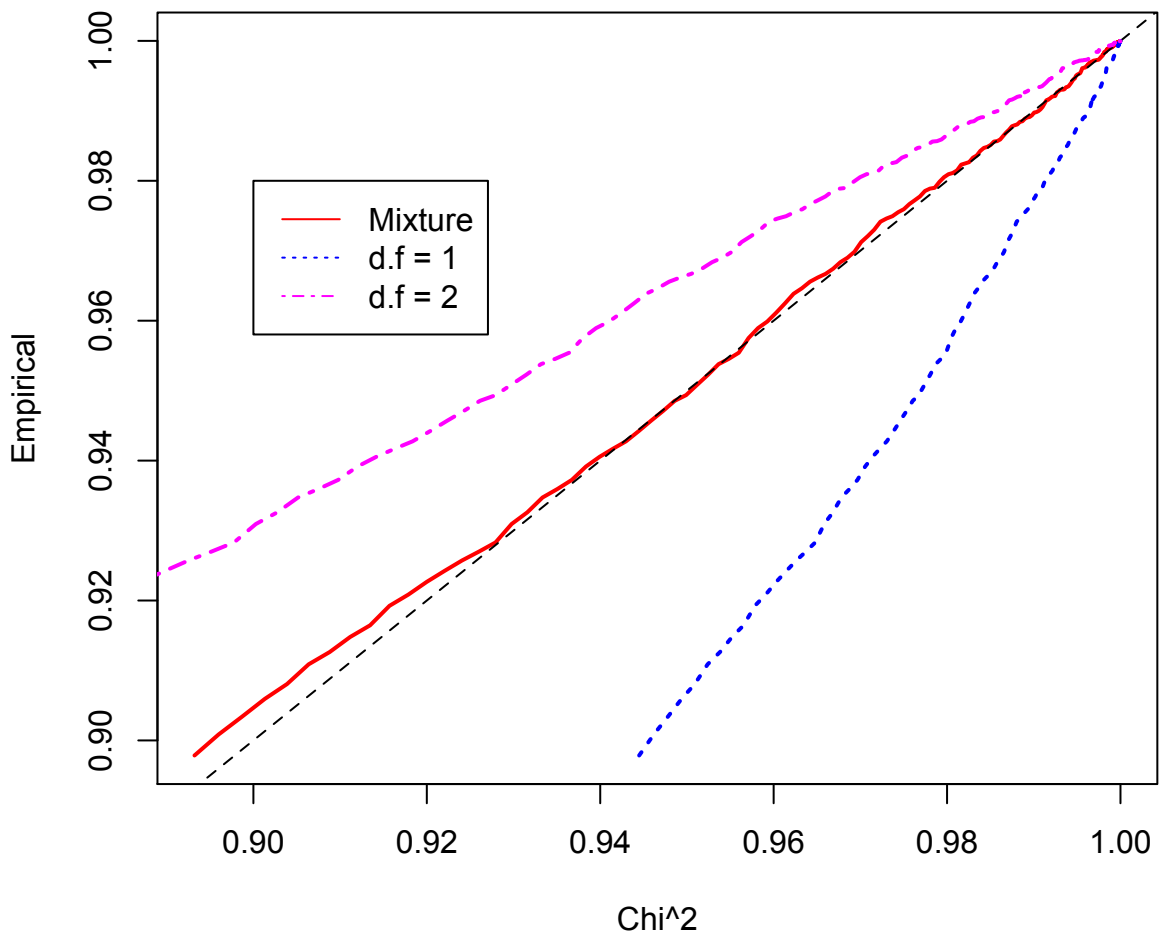

Supplement: Figure S1 — Quantile–Quantile plot of three asymptotic distributions (x-axis) for the MEME LRT test versus the LRT derived by parametric bootstrap (y-axis), limited to the meaningful test p-value range of . The distribution is too liberal (lying below the line), the is too conservative, while the mixture is approximately correct. (PDF) [file pgen.1002764.s001.pdf]
