# Supplementary material for: Detecting Individual Sites Subject to Episodic Diversifying Selection
Source: PLoS Genet. 2012 Jul 12;8(7):e1002764. doi: 10.1371/journal.pgen.1002764 (PMC3395634; doi:10.1371/journal.pgen.1002764)
Supplement: Figure S2 — Simulation parameters for generating datasets for evaluating the empirical Bayes inference of branch-site combinations under selection. Branches are colored according the the value of used to evolve sequences along them; branches simulated under positive selection are also labeled with values. (PDF) [file pgen.1002764.s002.pdf]

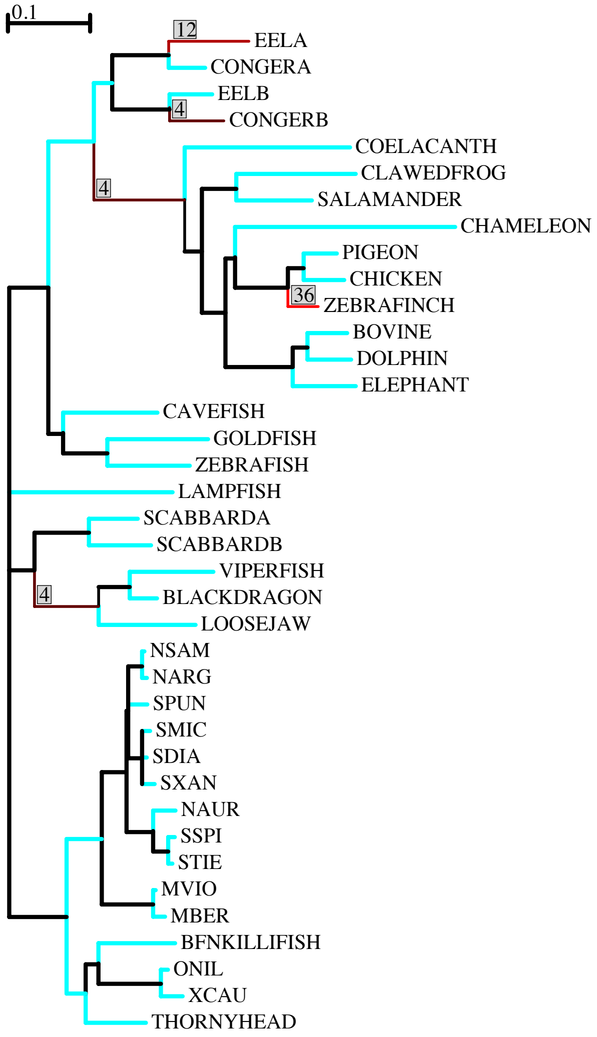

50% of sites.
Strongly negatively selected background (ω = 0.1)

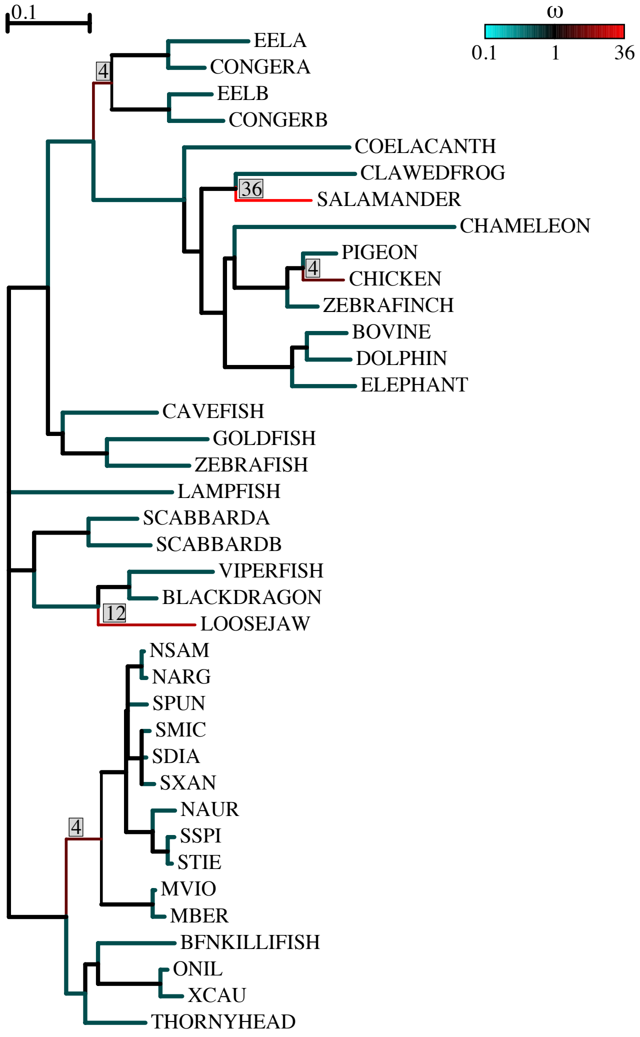

30% of sites.
Weakly negatively selected background (ω = 0.5)

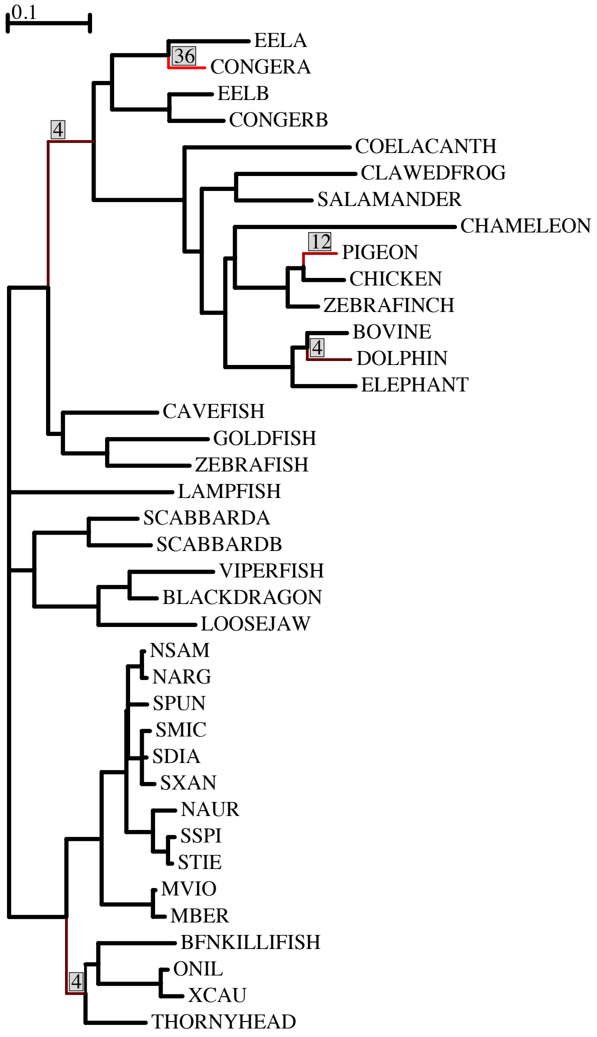

20% of sites.
Neutral background (ω = 1)
