# Supplementary material for: Detecting Individual Sites Subject to Episodic Diversifying Selection
Source: PLoS Genet. 2012 Jul 12;8(7):e1002764. doi: 10.1371/journal.pgen.1002764 (PMC3395634; doi:10.1371/journal.pgen.1002764)
Supplement: Figure S3 — Summary of empirical Bayes inference of branches under selection on data simulated using the selective parameters from Figure S2. Each branch is colored according to the proportion of times it was found to have an empirical Bayes factor of 20 or greater at sites with MEME p-value of 0.05 or less. Branches with detection rates are also labeled with the values of the rates. (PDF) [file pgen.1002764.s003.pdf]

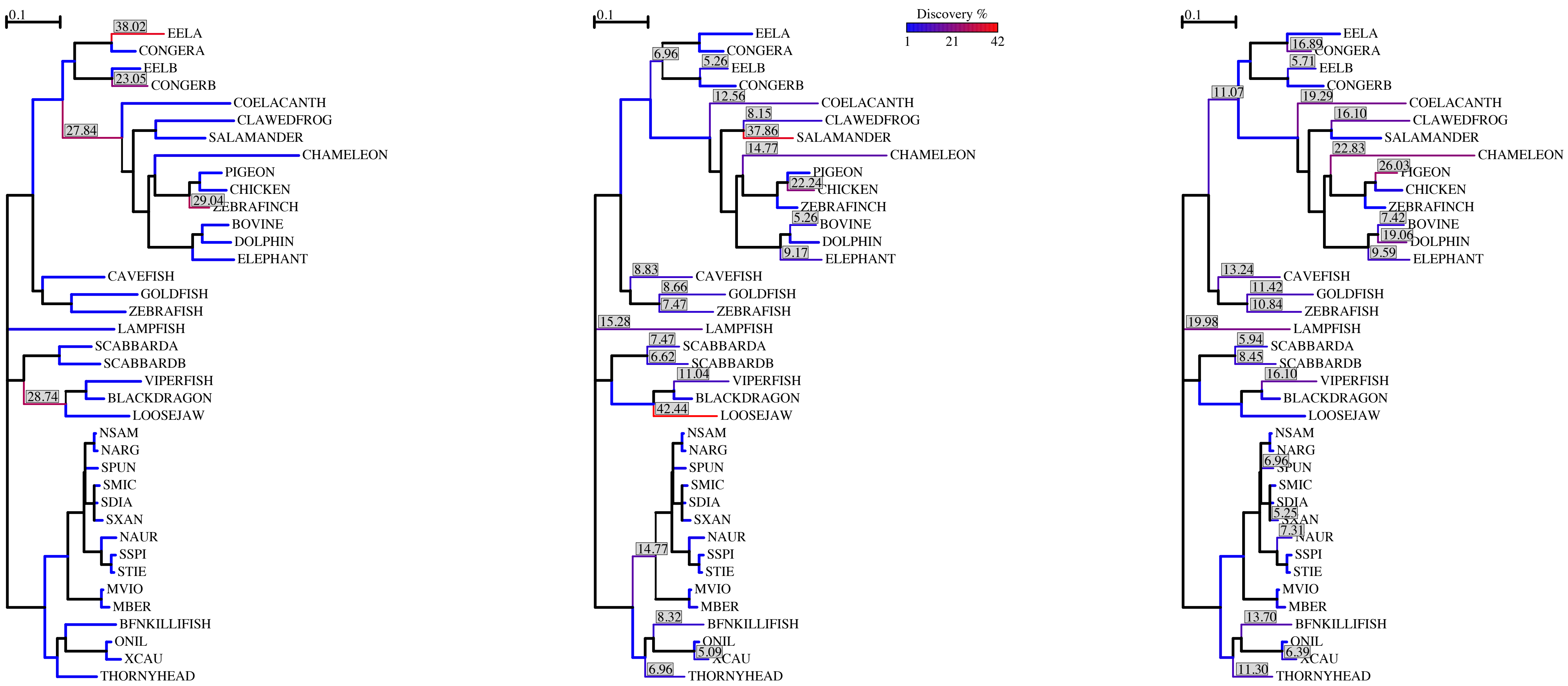

50% of sites.
Strongly negatively selected background (ω = 0.1)

30% of sites.
Weakly negatively selected background (ω = 0.5)

20% of sites.
Neutral background (ω = 1)
