# Supplementary material for: Detecting Individual Sites Subject to Episodic Diversifying Selection
Source: PLoS Genet. 2012 Jul 12;8(7):e1002764. doi: 10.1371/journal.pgen.1002764 (PMC3395634; doi:10.1371/journal.pgen.1002764)
Supplement: Table S1 — False positive rates for data sets simulated under strict neutrality using empirical trees from TreeBase. The entries are sorted in order of increasing mean false positive rate derived from simulated data (10 replicates per tree). Mean divergence between any pair of leaves in a given tree is reported in expected nucleotide substitutions per site. False positive range reports the minimum and maximum values for false positive rates for an individual replicate. 95% confidence intervals are derived from the binomial distribution with the probability of success , and the number of trials equal to the number of codons. This range provides the expected spread of per replicate false positive rates for a test that has the probability of making a false positive error of exactly over tests. (PDF) [file pgen.1002764.s004.pdf]

| | | | | Rates of false positives / replicate, % | | |
|---|---|---|---|---|---|---|
| TreeBase ID | Sequences | Codons | Mean divergence | Mean | Range | 95% Confidence Interval |
| 51737 | 96 | 209 | 0.0404 | 0.191 | 0 – 0.478 | 2.39 – 8.13 |
| 47706 | 97 | 298 | 0.0148 | 0.268 | 0 – 0.671 | 2.68 – 7.72 |
| 26258 | 158 | 78 | 0.0139 | 0.385 | 0 – 2.56 | 1.28 – 10.3 |
| 27576 | 155 | 218 | 0.00874 | 0.459 | 0 – 1.83 | 2.29 – 8.26 |
| 50407 | 112 | 233 | 0.027 | 0.515 | 0 – 1.29 | 2.58 – 8.15 |
| 50250 | 147 | 912 | 0.0271 | 0.537 | 0.219 – 1.43 | 3.62 – 6.47 |
| 47670 | 97 | 231 | 0.0392 | 0.693 | 0 – 1.3 | 2.6 – 8.23 |
| 47707 | 131 | 222 | 0.0416 | 0.991 | 0.45 – 1.35 | 2.25 – 8.11 |
| 47708 | 133 | 176 | 0.0503 | 1.65 | 0 – 3.98 | 2.27 – 8.52 |
| 43786 | 190 | 467 | 0.109 | 1.99 | 1.07 – 2.57 | 3.21 – 7.07 |
| 51735 | 102 | 217 | 0.181 | 2.03 | 0.922 – 3.69 | 2.3 – 8.29 |
| 26268 | 101 | 216 | 0.0637 | 2.13 | 0.463 – 3.24 | 2.31 – 8.33 |
| 20793 | 139 | 155 | 0.0552 | 2.39 | 0.645 – 4.52 | 1.94 – 8.39 |
| 43799 | 109 | 216 | 0.124 | 2.92 | 1.39 – 4.63 | 2.31 – 7.87 |
| 50066 | 191 | 279 | 0.0868 | 3.33 | 1.08 – 5.73 | 2.51 – 7.53 |
| 31473 | 177 | 467 | 0.113 | 4.07 | 2.36 – 5.78 | 3 – 7.07 |
| 49597 | 191 | 210 | 0.155 | 4.33 | 2.38 – 6.19 | 2.38 – 8.1 |
| 50488 | 100 | 157 | 0.158 | 4.39 | 2.55 – 6.37 | 1.91 – 8.92 |
| 47638 | 110 | 233 | 0.286 | 4.68 | 2.15 – 6.87 | 2.58 – 8.15 |
| 48737 | 146 | 193 | 0.0777 | 4.72 | 3.11 – 6.74 | 2.07 – 8.29 |
| 31472 | 160 | 217 | 0.114 | 4.93 | 3.23 – 7.37 | 2.3 – 8.29 |
| 31078 | 156 | 213 | 0.195 | 5.02 | 2.82 – 6.57 | 2.35 – 7.98 |
| 50259 | 167 | 216 | 0.509 | 5.05 | 1.85 – 7.87 | 2.31 – 7.87 |
| 49966 | 153 | 215 | 0.14 | 5.12 | 4.19 – 6.98 | 2.33 – 8.37 |
| 7471 | 103 | 569 | 0.459 | 5.29 | 2.99 – 6.33 | 3.34 – 6.85 |
| 48654 | 169 | 351 | 0.402 | 5.3 | 2.85 – 7.98 | 2.85 – 7.41 |
| 44942 | 190 | 203 | 0.351 | 5.32 | 2.46 – 8.87 | 2.46 – 8.37 |
| 50998 | 108 | 234 | 0.215 | 5.34 | 2.56 – 8.12 | 2.56 – 8.12 |
| 26494 | 121 | 199 | 0.753 | 5.38 | 2.01 – 10.1 | 2.01 – 8.04 |
| 25455 | 137 | 299 | 0.277 | 5.52 | 3.34 – 8.03 | 2.68 – 7.36 |
| 45979 | 142 | 208 | 0.185 | 5.53 | 1.92 – 9.13 | 2.4 – 8.17 |
| 9873 | 171 | 469 | 0.298 | 5.76 | 4.69 – 7.25 | 3.2 – 7.04 |
| 31295 | 104 | 231 | 0.406 | 5.8 | 3.9 – 7.36 | 2.16 – 7.79 |
| 29855 | 160 | 210 | 0.359 | 5.81 | 4.76 – 8.57 | 2.38 – 8.1 |
| 50442 | 104 | 545 | 0.41 | 5.87 | 4.59 – 9.36 | 3.3 – 6.79 |
| 48655 | 169 | 221 | 0.637 | 6.43 | 2.71 – 10.4 | 2.26 – 8.14 |
