# Supplementary material for: Detecting Individual Sites Subject to Episodic Diversifying Selection
Source: PLoS Genet. 2012 Jul 12;8(7):e1002764. doi: 10.1371/journal.pgen.1002764 (PMC3395634; doi:10.1371/journal.pgen.1002764)
Supplement: Table S2 — False positive rates for three empirical trees from TreeBase when the parameters of the null model are varied: 20% of the branches are simulated with the foreground , and the remainder under the background . 10 replicates with codons each per tree- pair were simulated. The synonymous rate was set to for the first codons, for the next codons, and for the last codons. (PDF) [file pgen.1002764.s005.pdf]

		TreeBase tree		
		Tr25302	Tr25621	Tr25483
Mean pairwise divergence		0.30	0.40	0.04
ω		False Positive Rate at p = 0.05		
Background	Foreground			
0.1	0.25	0	0.0067	0.0033
0.1	0.5	0.0033	0.0033	0
0.1	0.75	0.017	0.02	0.013
0.1	1.0	0.013	0.0067	0.01
0.25	0.5	0.013	0.01	0.0033
0.25	0.75	0.017	0.0067	0.0033
0.25	1.0	0.023	0.017	0.017
0.5	0.75	0.01	0.027	0.01
0.5	1.0	0.027	0.017	0.01
0.75	1.0	0.063	0.03	0.013
Prior predictive p (uniform prior)		0.019	0.0143	0.008
