# Supplementary material for: Detecting Individual Sites Subject to Episodic Diversifying Selection
Source: PLoS Genet. 2012 Jul 12;8(7):e1002764. doi: 10.1371/journal.pgen.1002764 (PMC3395634; doi:10.1371/journal.pgen.1002764)
Supplement: Table S3 — Comparative performance of FEL and MEME on simulated data where does not vary among tree branches. The rate of false positives (FP) and power are reported for a fixed nominal test p-value of . Power is also shown for the p-value that achieves FP of 0.05, estimated empirically from the distribution of p-values on the subset of sites evolving neutrally. (PDF) [file pgen.1002764.s006.pdf]

Simulation	FP : Power		Power at FP= 0.05	
	FEL	MEME	FEL	MEME
Encephalitis virus env				
$\omega^+ = 1.25$	0.01:0.03	0.01:0.04	0.04	0.10
$\omega^+ = 1.5$	0.00:0.03	0.01:0.03	0.09	0.14
$\omega^+ = 1.75$	0.00:0.03	0.01:0.03	0.08	0.10
$\omega^+ = 2$	0.00:0.05	0.01:0.06	0.13	0.17
$\omega^+ = 3$	0.00:0.09	0.01:0.09	0.19	0.23
$\omega^+ = 5$	0.00:0.19	0.01:0.18	0.34	0.43
$\omega^+ = 8$	0.00:0.28	0.00:0.26	0.50	0.59
$\omega^+ = 12$	0.00:0.34	0.00:0.31	0.54	0.71
$\omega^+ = 16$	0.00:0.38	0.00:0.34	0.63	0.77
Vertebrate Rhodopsin				
$\omega^+ = 1.25$	0.01:0.07	0.02:0.08	0.07	0.09
$\omega^+ = 1.5$	0.01:0.08	0.02:0.09	0.08	0.13
$\omega^+ = 1.75$	0.01:0.13	0.02:0.12	0.14	0.17
$\omega^+ = 2$	0.01:0.19	0.02:0.19	0.13	0.18
$\omega^+ = 3$	0.01:0.32	0.02:0.32	0.34	0.37
$\omega^+ = 5$	0.01:0.48	0.02:0.45	0.51	0.55
$\omega^+ = 8$	0.01:0.67	0.01:0.67	0.74	0.76
$\omega^+ = 12$	0.00:0.71	0.01:0.69	0.80	0.81
$\omega^+ = 16$	0.00:0.76	0.01:0.73	0.88	0.89
Camelid VHH				
$\omega^+ = 1.25$	0.01:0.11	0.02:0.12	0.06	0.13
$\omega^+ = 1.5$	0.02:0.19	0.02:0.23	0.14	0.21
$\omega^+ = 1.75$	0.01:0.34	0.02:0.32	0.26	0.30
$\omega^+ = 2$	0.01:0.51	0.02:0.47	0.48	0.51
$\omega^+ = 3$	0.01:0.74	0.02:0.73	0.64	0.69
$\omega^+ = 5$	0.01:0.93	0.02:0.91	0.93	0.93
$\omega^+ = 8$	0.01:0.98	0.01:0.97	0.98	0.99
$\omega^+ = 12$	0.01:0.97	0.01:0.97	0.97	0.98
$\omega^+ = 16$	0.02:0.99	0.03:0.99	0.99	0.99
