# Supplementary material for: Detecting Individual Sites Subject to Episodic Diversifying Selection
Source: PLoS Genet. 2012 Jul 12;8(7):e1002764. doi: 10.1371/journal.pgen.1002764 (PMC3395634; doi:10.1371/journal.pgen.1002764)
Supplement: Table S4 — Positively selected sites in abalone sperm lysin. stands for a positively selected site and stands for a negatively selected site (FEL ). and reflect borderline significant sites (FEL p between and ). and denote significant sites (FEL ). (PDF) [file pgen.1002764.s007.pdf]

			MEME MLE			FEL MLE		p-value		q-value	log L		
Site	α	β^-	q^-	β^+	q^+	α	β	MEME	FEL	MEME	MEME	FEL	FEL result
6*[a]	0.00	0.00	0.79	23.89	0.21	0.94	2.60	0.01	0.27	0.06	-39.08	-43.00	+
10*	1.85	1.85	0.96	483.52	0.04	0.46	3.28	0.01	0.26	0.06	-56.29	-59.77	+
14*	0.68	0.00	0.78	20.00	0.22	1.76	1.89	0.00	0.94	0.06	-54.58	-59.09	+
15	0.00	0.00	0.89	15.97	0.11	0.00	1.34	0.00	0.18	0.04	-24.16	-28.70	+
16	0.00	0.00	0.97	11.87	0.03	0.00	0.30	0.02	0.38	0.16	-12.35	-15.29	+
36*	0.00	0.00	0.61	11.09	0.39	0.19	2.70	0.00	0.02	0.06	-50.11	-51.78	+ + +
37*	0.97	0.00	0.83	16.35	0.17	0.95	1.43	0.00	0.61	0.06	-41.20	-45.56	+
41*	0.00	0.00	0.58	8.80	0.42	0.14	2.37	0.01	0.07	0.08	-51.23	-53.38	++
44*	0.00	0.00	0.65	9.64	0.35	0.00	2.45	0.00	0.01	0.02	-50.60	-53.51	+ + +
45*	0.00	0.00	0.50	3.59	0.50	0.00	1.63	0.03	0.04	0.21	-29.50	-29.99	+ + +
68*	0.00	0.00	0.88	25.42	0.12	0.00	1.90	0.00	0.40	0.03	-32.89	-38.51	+
75*	0.00	0.00	0.63	4.69	0.37	0.00	1.76	0.01	0.01	0.06	-36.05	-37.05	+ + +
83*	2.55	0.00	0.68	15.49	0.32	2.73	2.76	0.01	0.99	0.07	-61.60	-65.92	+
87*	0.33	0.00	0.70	15.97	0.30	0.05	4.66	0.00	0.01	0.00	-53.46	-58.45	+ + +
106*	0.00	0.00	0.79	11.91	0.21	0.00	1.94	0.00	0.01	0.03	-34.75	-37.01	+ + +
107*	0.96	0.35	0.93	997.29	0.07	1.60	1.70	0.00	0.95	0.01	-43.22	-52.86	+
108*	1.43	0.00	0.93	18.81	0.07	1.76	0.59	0.04	0.21	0.26	-27.82	-32.32	−
109*	0.00	0.00	0.87	6.41	0.13	0.00	0.55	0.04	0.30	0.25	-23.37	-25.16	+
113*	0.00	0.00	0.61	10.45	0.39	0.00	3.06	0.00	0.09	0.05	-53.47	-57.04	++
119*	1.09	0.00	0.76	10.61	0.24	1.28	1.54	0.01	0.79	0.08	-53.24	-56.98	+
123*	0.00	0.00	0.89	32.30	0.11	0.00	1.49	0.00	0.03	0.00	-32.72	-40.93	+ + +
125*	1.22	0.00	0.91	20.39	0.09	1.52	0.68	0.05	0.28	0.25	-38.92	-42.35	−
126*	0.37	0.00	0.68	9.09	0.32	0.37	2.49	0.01	0.03	0.06	-45.48	-47.41	+ + +
130*	0.00	0.00	0.96	9.32	0.04	0.00	0.30	0.01	0.21	0.10	-12.16	-14.90	+
131	0.00	0.00	0.43	2.47	0.57	0.00	1.39	0.05	0.04	0.25	-25.62	-25.89	+ + +
132 *	0.00	0.00	0.76	34.99	0.24	0.93	2.69	0.02	0.28	0.11	-37.93	-40.63	+

[a]* – a surface (exposed) residue, as designated in Yang and Swanson (2002)
