# Supplementary material for: Detecting Individual Sites Subject to Episodic Diversifying Selection
Source: PLoS Genet. 2012 Jul 12;8(7):e1002764. doi: 10.1371/journal.pgen.1002764 (PMC3395634; doi:10.1371/journal.pgen.1002764)
Supplement: Table S5 — Positively selected sites in camelid VHH. stands for a positively selected site and stands for a negatively selected site (FEL ). and reflect borderline significant sites (FEL p between and ). and denote significant sites (FEL ). (PDF) [file pgen.1002764.s008.pdf]

	MEME MLE					FEL MLE		p-value		q-value	log L		
Site	α	β^-	q^-	β^+	q^+	α	β	MEME	FEL	MEME	MEME	FEL	FEL result
5	0.00	0.00	0.99	21.94	0.01	0.00	0.09	0.001	0.306	0.01	-21.36	-26.80	+
10	0.43	0.04	0.94	5.23	0.06	0.46	0.30	0.014	0.421	0.06	-88.21	-93.28	−
14	0.49	0.43	0.33	2.13	0.67	0.49	1.50	0.002	0.001	0.02	-258.48	-258.44	+++
23	0.56	0.56	0.00	1.78	1.00	0.56	1.77	0.001	0.000	0.01	-242.08	-242.31	+++
24	0.47	0.40	0.93	7.99	0.07	0.51	0.78	0.028	0.306	0.10	-142.09	-144.43	+
25	0.37	0.37	0.97	20.44	0.03	0.41	0.75	0.000	0.162	0.00	-137.31	-145.78	+
28	1.13	0.71	0.82	8.38	0.18	1.24	1.72	0.005	0.283	0.03	-290.45	-294.24	+
29	1.48	0.98	0.77	9.22	0.23	1.56	2.24	0.009	0.294	0.04	-313.55	-316.85	+
30	1.12	0.00	0.50	4.16	0.50	1.23	1.89	0.003	0.220	0.02	-290.80	-295.29	+
32	1.03	0.82	0.85	13.89	0.15	1.14	2.00	0.000	0.091	0.00	-282.10	-288.58	++
33	1.20	1.20	0.65	15.33	0.35	1.15	4.37	0.000	0.000	0.00	-417.89	-421.35	+++
35	1.63	1.24	0.95	49.65	0.05	1.91	1.91	0.006	0.998	0.03	-304.14	-309.07	−
38	1.32	0.02	0.99	451.80	0.01	1.46	0.11	0.007	0.000	0.04	-107.46	-114.40	−−−
40	0.17	0.00	0.57	2.73	0.43	0.18	1.10	0.000	0.000	0.00	-161.27	-162.85	+++
45	1.16	0.00	0.94	13.48	0.06	1.19	0.34	0.020	0.001	0.08	-155.58	-160.53	−−−
47	1.13	0.94	0.87	10.56	0.13	1.10	1.68	0.043	0.253	0.14	-269.74	-270.92	+
50	0.96	0.26	0.73	14.61	0.27	1.16	2.46	0.000	0.029	0.00	-223.17	-231.39	+++
51	1.77	0.87	0.93	78.83	0.07	2.20	1.77	0.000	0.416	0.00	-319.15	-336.07	−
52	2.83	0.92	0.81	16.40	0.19	3.03	2.44	0.002	0.427	0.02	-394.70	-404.91	−
53	2.10	1.45	0.80	15.89	0.20	2.35	3.00	0.009	0.386	0.04	-388.44	-391.86	+
54	1.18	1.18	0.72	11.28	0.28	1.14	3.11	0.000	0.001	0.00	-381.61	-387.42	+++
55	1.35	1.35	0.90	16.20	0.10	1.34	2.22	0.007	0.071	0.04	-309.51	-312.19	++
56	1.74	0.84	0.80	9.75	0.20	1.82	2.06	0.017	0.647	0.07	-292.04	-295.47	+
67	0.37	0.00	0.99	58.55	0.01	0.37	0.12	0.032	0.079	0.11	-70.71	-73.48	−−
68	0.15	0.00	0.96	7.17	0.04	0.15	0.18	0.004	0.856	0.02	-47.92	-53.85	+
72	0.50	0.46	0.93	69.05	0.07	1.09	1.16	0.000	0.877	0.00	-245.26	-270.83	+
75	0.62	0.58	0.98	74.85	0.02	0.70	0.83	0.001	0.650	0.01	-175.98	-182.09	+
80	0.73	0.00	0.75	5.56	0.25	0.80	1.15	0.000	0.358	0.00	-178.17	-184.45	+
83	0.00	0.00	0.96	11.15	0.04	0.00	0.16	0.023	0.864	0.09	-63.14	-66.08	+
92	0.06	0.05	0.99	188.42	0.01	0.13	0.13	0.038	0.966	0.13	-44.61	-47.08	−
