# Supplementary material for: Detecting Individual Sites Subject to Episodic Diversifying Selection
Source: PLoS Genet. 2012 Jul 12;8(7):e1002764. doi: 10.1371/journal.pgen.1002764 (PMC3395634; doi:10.1371/journal.pgen.1002764)
Supplement: Table S6 — Positively selected sites in Diatom silicon transporters found by MEME at . The FEL result column summarizes the classification obtained by FEL. stands for a positively selected site and stands for a negatively selected site (FEL ). and reflect borderline significant sites (FEL p between and ). and denote significant sites (FEL ). (PDF) [file pgen.1002764.s009.pdf]

	MEME MLE					FEL MLE		p-value		q-value	log L		
Site	α	β^-	q^-	β^+	q^+	α	β	MEME	FEL	MEME	MEME	FEL	FEL result
12	0.89	0.11	0.98	58.53	0.02	0.98	0.20	0.044	0.000	0.31	-114.59	-119.12	− − −
14	0.70	0.03	0.98	27.04	0.02	0.80	0.08	0.050	0.000	0.31	-84.76	-87.10	− − −
16	1.42	0.00	0.90	8.71	0.10	1.53	0.31	0.038	0.000	0.30	-123.87	-132.15	− − −
24	1.35	0.03	0.96	103.08	0.04	1.77	0.10	0.008	0.000	0.10	-126.09	-130.55	− − −
32	0.69	0.03	0.98	9.85	0.02	0.75	0.12	0.041	0.000	0.30	-71.06	-77.67	− − −
37	0.55	0.06	0.97	6.56	0.03	0.58	0.14	0.044	0.006	0.31	-82.33	-87.09	− − −
45	0.90	0.00	0.95	105.59	0.05	1.39	0.23	0.000	0.001	0.00	-92.65	-115.44	− − −
50	0.10	0.04	0.99	7.77	0.01	0.15	0.09	0.026	0.518	0.22	-43.01	-46.82	−
61	0.81	0.00	0.90	4.33	0.10	0.81	0.31	0.005	0.009	0.06	-123.90	-137.56	− − −
64	1.35	0.19	0.81	6.95	0.19	1.34	0.89	0.024	0.211	0.21	-200.04	-207.11	−
67	0.46	0.00	0.96	5.12	0.04	0.47	0.11	0.045	0.020	0.31	-54.97	-62.33	− − −
73	1.04	0.15	0.96	252.44	0.04	1.20	0.33	0.014	0.000	0.14	-161.70	-168.36	− − −
82	1.36	0.15	0.95	16.53	0.05	1.37	0.32	0.046	0.000	0.31	-160.87	-165.78	− − −
83	0.85	0.04	0.93	10.55	0.07	0.98	0.26	0.011	0.000	0.12	-124.47	-133.25	− − −
85	1.20	0.02	0.97	11.77	0.03	1.19	0.15	0.016	0.000	0.16	-126.37	-136.66	− − −
90	1.19	0.16	0.95	72.06	0.05	2.15	0.41	0.001	0.001	0.01	-143.51	-155.86	− − −
91	0.77	0.07	0.96	38.02	0.04	0.78	0.22	0.001	0.004	0.02	-123.14	-131.57	− − −
94	1.00	0.03	0.96	74.71	0.04	1.45	0.21	0.002	0.000	0.03	-118.39	-132.71	− − −
97	1.28	0.13	0.92	59.21	0.08	1.96	0.44	0.000	0.000	0.01	-199.47	-214.68	− − −
99	0.68	0.06	0.93	16.57	0.07	0.71	0.34	0.001	0.170	0.02	-107.60	-118.88	−
106	0.73	0.11	0.96	24.71	0.04	0.83	0.29	0.042	0.007	0.31	-129.47	-134.73	− − −
113	0.67	0.03	0.99	32.47	0.01	0.66	0.09	0.002	0.000	0.03	-89.35	-98.35	− − −
116	0.44	0.04	0.95	74.16	0.05	0.64	0.34	0.000	0.100	0.00	-132.68	-150.85	− −
124	0.97	0.04	0.86	10.45	0.14	1.08	0.57	0.001	0.067	0.01	-176.19	-187.99	− −
133	1.76	0.01	0.95	177.59	0.05	3.59	0.20	0.001	0.000	0.02	-94.14	-122.91	− − −
140	0.93	0.09	0.84	10.44	0.16	1.03	0.75	0.000	0.382	0.01	-192.22	-205.78	−
142	0.30	0.10	0.97	339.98	0.03	0.47	0.21	0.002	0.083	0.03	-112.78	-120.05	− −
144	0.90	0.09	0.96	12.36	0.04	0.94	0.22	0.048	0.000	0.31	-123.74	-127.82	− − −
154	0.01	0.01	0.99	81.44	0.01	0.00	0.09	0.000	0.663	0.00	-28.02	-45.37	+
155	1.59	0.12	0.92	53.16	0.08	1.68	0.43	0.003	0.000	0.04	-180.42	-191.73	− − −
166	1.39	0.00	0.99	46.08	0.01	1.51	0.04	0.021	0.000	0.21	-97.17	-104.72	− − −
169	1.14	0.06	0.95	1466.39	0.05	1.20	0.26	0.000	0.000	0.00	-150.17	-166.65	− − −
181	1.12	0.00	0.94	19.31	0.06	1.24	0.30	0.000	0.000	0.01	-126.59	-148.52	− − −
193	0.44	0.00	0.88	4.18	0.12	0.61	0.30	0.001	0.207	0.02	-104.94	-118.80	−
197	0.64	0.25	0.98	38.25	0.02	0.88	0.30	0.048	0.043	0.30	-116.77	-119.37	− − −
198	0.31	0.00	0.99	31.98	0.01	0.31	0.03	0.024	0.005	0.22	-38.66	-41.91	− − −
212	1.19	0.06	0.93	23.65	0.07	1.25	0.32	0.010	0.001	0.12	-132.84	-140.33	− − −
213	0.74	0.07	0.97	29.03	0.03	0.74	0.19	0.001	0.002	0.02	-110.78	-121.21	− − −
222	1.41	0.01	0.99	65.02	0.01	1.41	0.09	0.034	0.000	0.28	-101.22	-108.52	− − −
237	0.74	0.08	0.94	144.41	0.06	1.15	0.42	0.000	0.006	0.00	-176.68	-210.14	− − −
242	1.56	0.20	0.91	31.62	0.09	2.27	0.57	0.000	0.001	0.01	-197.33	-212.97	− − −
243	0.36	0.00	0.99	214.06	0.01	0.36	0.02	0.022	0.001	0.21	-31.86	-35.08	− − −
273	0.73	0.00	0.98	2346.33	0.02	1.05	0.06	0.000	0.000	0.01	-64.00	-73.69	− − −
284	0.63	0.01	0.84	29.64	0.16	0.78	1.03	0.000	0.532	0.00	-170.17	-210.50	+
288	0.39	0.13	0.95	18.93	0.05	0.46	0.39	0.001	0.738	0.02	-108.45	-116.35	−
291	1.08	0.10	0.92	47.14	0.08	1.31	0.57	0.025	0.083	0.21	-148.17	-157.68	− −
295	1.13	0.00	0.99	27.86	0.01	1.13	0.03	0.036	0.000	0.28	-50.03	-56.82	− − −
296	0.59	0.10	0.97	34.56	0.03	0.63	0.24	0.009	0.016	0.10	-125.77	-132.40	− − −
