# Supplementary material for: Detecting Individual Sites Subject to Episodic Diversifying Selection
Source: PLoS Genet. 2012 Jul 12;8(7):e1002764. doi: 10.1371/journal.pgen.1002764 (PMC3395634; doi:10.1371/journal.pgen.1002764)
Supplement: Table S7 — Positively selected sites in Drosophila adh found by MEME at . The FEL result column summarizes the classification obtained by FEL. stands for a positively selected site and stands for a negatively selected site (FEL ). and reflect borderline significant sites (FEL p between and ). and denote significant sites (FEL ). (PDF) [file pgen.1002764.s010.pdf]

	MEME MLE					FEL MLE		p-value		q-value	log L		
Site	α	β^-	q^-	β^+	q^+	α	β	MEME	FEL	MEME	MEME	FEL	FEL result
6	0.00	0.00	0.95	8.99	0.05	0.00	0.30	0.049	0.466	1.00	-12.48	-15.24	$+$
46	0.61	0.00	0.97	428.58	0.03	0.91	0.26	0.017	0.191	0.74	-17.18	-22.32	$-$
82	0.00	0.00	0.94	41.94	0.06	0.00	0.48	0.007	0.214	0.57	-16.96	-20.33	$+$
133	0.21	0.00	0.84	17.67	0.16	0.22	0.68	0.045	0.286	1.00	-28.88	-30.61	$+$
163	0.61	0.37	0.82	19.49	0.18	0.80	1.63	0.019	0.442	0.69	-41.79	-44.63	$+$
165	0.00	0.00	0.82	13.63	0.18	0.14	0.87	0.013	0.174	0.68	-29.24	-31.74	$+$
166	0.97	0.00	0.97	26.57	0.03	0.95	0.26	0.034	0.226	1.00	-23.94	-28.59	$-$
170	0.71	0.12	0.91	40.10	0.09	0.70	0.64	0.010	0.917	0.65	-36.24	-40.02	$-$
197	0.00	0.00	0.81	11.83	0.19	0.00	0.93	0.003	0.016	0.64	-26.49	-28.71	$+++$
227	0.00	0.00	0.97	40.73	0.03	0.29	0.31	0.005	0.964	0.59	-11.86	-16.97	$+$
