# Supplementary material for: Detecting Individual Sites Subject to Episodic Diversifying Selection
Source: PLoS Genet. 2012 Jul 12;8(7):e1002764. doi: 10.1371/journal.pgen.1002764 (PMC3395634; doi:10.1371/journal.pgen.1002764)
Supplement: Table S9 — Positively selected sites in Flavivirus NS5. stands for a positively selected site and stands for a negatively selected site (FEL ). and reflect borderline significant sites (FEL p between and ). and denote significant sites (FEL ). (PDF) [file pgen.1002764.s012.pdf]

	MEME MLE					FEL MLE		p-value		q-value	log L		
Site	α	β^-	q^-	β^+	q^+	α	β	MEME	FEL	MEME	MEME	FEL	FEL result
43	0.20	0.00	0.92	3.42	0.08	0.21	0.07	0.030	0.213	1.00	-26.26	-30.83	$-$
45	0.16	0.00	0.90	19.24	0.10	0.38	0.08	0.021	0.049	1.00	-40.23	-43.44	$---$
126	0.00	0.00	0.68	1.04	0.32	0.07	0.24	0.039	0.507	1.00	-37.44	-39.58	$+$
175	0.10	0.00	0.85	5.54	0.15	0.23	0.11	0.029	0.434	1.00	-32.87	-36.49	$-$
