# Supplementary material for: Detecting Individual Sites Subject to Episodic Diversifying Selection
Source: PLoS Genet. 2012 Jul 12;8(7):e1002764. doi: 10.1371/journal.pgen.1002764 (PMC3395634; doi:10.1371/journal.pgen.1002764)
Supplement: Table S10 — Positively selected sites in Hepatitis D virus Ag. stands for a positively selected site and stands for a negatively selected site (FEL ). and reflect borderline significant sites (FEL p between and ). and denote significant sites (FEL ). (PDF) [file pgen.1002764.s013.pdf]

	MEME MLE					FEL MLE		p-value		q-value	log L		
Site	α	β^-	q^-	β^+	q^+	α	β	MEME	FEL	MEME	MEME	FEL	FEL result
6	1.22	1.22	0.97	575.77	0.03	0.85	2.13	0.014	0.213	0.16	-52.24	-54.89	+
13	0.00	0.00	0.73	62.72	0.27	0.00	13.47	0.007	0.067	0.13	-15.65	-18.06	++
24	0.00	0.00	0.40	3.37	0.60	0.00	1.88	0.002	0.001	0.12	-40.01	-39.96	+ + +
28	1.38	0.07	0.88	32.06	0.12	1.94	1.87	0.033	0.957	0.32	-48.68	-51.37	−
31	0.00	0.00	0.98	19.17	0.02	0.00	0.33	0.008	0.245	0.12	-12.27	-15.83	+
35	0.00	0.00	0.98	24.44	0.02	0.00	0.31	0.004	0.256	0.10	-12.33	-16.33	+
90	0.26	0.16	0.98	66.05	0.02	0.38	0.60	0.003	0.685	0.10	-23.83	-28.76	+
99	0.00	0.00	0.98	1132.96	0.02	0.67	0.15	0.015	0.325	0.17	-11.45	-14.88	−
107	0.00	0.00	0.98	1437.97	0.02	0.67	0.14	0.012	0.298	0.18	-11.43	-15.09	−
117	0.67	0.67	0.95	160.10	0.05	0.80	1.43	0.005	0.494	0.10	-52.28	-56.31	+
122	0.00	0.00	0.52	8.50	0.48	0.00	3.19	0.007	0.032	0.14	-69.43	-71.27	+ + +
140	0.00	0.00	0.59	4.37	0.41	0.00	1.87	0.002	0.002	0.14	-34.17	-34.82	+ + +
142	0.00	0.00	0.73	4.37	0.27	0.00	0.98	0.014	0.031	0.17	-30.39	-31.53	+ + +
145	0.00	0.00	0.73	4.44	0.27	0.00	1.19	0.004	0.009	0.12	-30.53	-31.82	+ + +
150	0.00	0.00	0.74	5.14	0.26	0.00	1.45	0.001	0.002	0.06	-32.45	-34.30	+ + +
159	0.17	0.00	0.79	13.53	0.21	0.47	1.63	0.013	0.178	0.18	-43.63	-46.15	+
160	0.00	0.00	0.73	5.90	0.27	0.00	1.22	0.018	0.034	0.18	-34.06	-35.09	+ + +
163	0.00	0.00	0.98	6078.21	0.02	0.45	0.16	0.000	0.477	0.01	-12.06	-21.73	−
173	1.27	0.00	0.83	24.42	0.17	1.49	1.52	0.013	0.978	0.17	-54.30	-57.79	+
183	0.30	0.30	0.93	97.34	0.07	0.91	1.44	0.002	0.604	0.09	-42.90	-47.89	+
