# Supplementary material for: Detecting Individual Sites Subject to Episodic Diversifying Selection
Source: PLoS Genet. 2012 Jul 12;8(7):e1002764. doi: 10.1371/journal.pgen.1002764 (PMC3395634; doi:10.1371/journal.pgen.1002764)
Supplement: Table S11 — Positively selected sites in HIV-1 reverse transcriptase (rt). stands for a positively selected site and stands for a negatively selected site (FEL ). and reflect borderline significant sites (FEL p between and ). and denote significant sites (FEL ). (PDF) [file pgen.1002764.s014.pdf]

	MEME MLE					FEL MLE		p-value		q-value	log L		
Site	α	β^-	q^-	β^+	q^+	α	β	MEME	FEL	MEME	MEME	FEL	FEL result
35	0.18	0.00	0.72	6.92	0.28	0.24	1.82	0.000	0.006	0.01	-120.50	-123.64	+ + +
48	0.23	0.21	0.99	67.77	0.01	0.24	0.56	0.000	0.140	0.00	-106.53	-116.48	+
64	0.15	0.14	0.99	109.00	0.01	0.15	0.33	0.000	0.238	0.00	-75.53	-82.69	+
69*[a]	0.72	0.00	0.94	10.00	0.06	0.72	0.53	0.001	0.481	0.03	-140.39	-148.35	−
75*	0.73	0.09	0.99	31.26	0.01	0.79	0.31	0.002	0.053	0.04	-104.06	-112.08	− −
102	0.00	0.00	0.10	0.32	0.90	0.00	0.29	0.006	0.003	0.10	-58.62	-58.61	+ + +
103*	1.25	0.00	0.70	21.77	0.30	1.26	3.49	0.000	0.001	0.00	-601.48	-608.64	+ + +
104	1.76	0.12	1.00	258.43	0.00	1.76	0.21	0.041	0.000	0.49	-109.41	-113.91	− − −
121	0.89	0.00	0.96	18.87	0.04	0.90	0.44	0.031	0.165	0.39	-135.78	-139.20	−
122	1.30	1.07	0.99	255.69	0.01	1.33	1.38	0.000	0.911	0.00	-236.76	-244.79	+
138*	0.31	0.02	0.99	22.56	0.01	0.31	0.17	0.026	0.318	0.33	-67.10	-70.78	−
151*	1.00	0.00	0.99	77.12	0.01	0.99	0.42	0.000	0.036	0.00	-113.27	-130.34	− − −
162	1.42	0.25	0.93	31.90	0.07	1.46	1.29	0.000	0.722	0.00	-312.23	-329.05	−
163	0.89	0.00	1.00	65.44	0.00	0.89	0.05	0.007	0.000	0.11	-59.71	-67.16	− − −
165	0.08	0.08	0.00	0.52	1.00	0.08	0.52	0.024	0.015	0.32	-106.07	-106.11	+ + +
181*	2.71	0.14	0.98	73.02	0.02	2.70	0.60	0.000	0.000	0.00	-152.20	-168.14	− − −
184*	0.03	0.03	0.85	12.66	0.15	3.41	1.01	0.011	0.253	0.16	-264.90	-272.98	−
188*	0.14	0.00	0.99	98.56	0.01	0.14	0.40	0.000	0.272	0.00	-65.21	-85.19	+
190*	1.32	0.00	0.95	21.32	0.05	1.53	0.48	0.006	0.002	0.10	-207.52	-213.04	− − −
200	0.23	0.23	0.00	2.34	1.00	0.23	2.33	0.000	0.000	0.00	-297.77	-297.91	+ + +
207	2.06	0.00	0.78	16.31	0.22	2.17	2.41	0.002	0.712	0.04	-344.68	-349.50	+
211	0.78	0.78	0.00	2.11	1.00	0.78	2.09	0.001	0.000	0.02	-312.84	-312.93	+ + +
213	3.33	0.00	1.00	8820.96	0.00	3.42	0.03	0.010	0.000	0.16	-150.83	-155.48	− − −
215*	0.57	0.00	0.98	75.10	0.02	0.57	0.41	0.000	0.465	0.00	-118.46	-138.17	−
228	1.42	0.05	0.99	165.25	0.01	1.66	0.25	0.000	0.000	0.00	-141.08	-156.14	− − −
245	0.36	0.36	0.71	14.11	0.29	0.40	3.28	0.000	0.000	0.00	-406.70	-411.28	+ + +
248	0.09	0.09	0.00	1.14	1.00	0.09	1.14	0.043	0.030	0.50	-100.45	-100.39	+ + +
272	0.13	0.00	0.90	89.62	0.10	0.19	1.76	0.000	0.003	0.00	-138.33	-145.41	+ + +
286	0.49	0.49	0.00	2.08	1.00	0.49	2.07	0.011	0.006	0.15	-105.80	-105.86	+ + +

[a] Substitutions at codons marked with * are associated with the development of drug resistance to RT inhibitors (Johnson et al., 2010).
