# Supplementary material for: Detecting Individual Sites Subject to Episodic Diversifying Selection
Source: PLoS Genet. 2012 Jul 12;8(7):e1002764. doi: 10.1371/journal.pgen.1002764 (PMC3395634; doi:10.1371/journal.pgen.1002764)
Supplement: Table S12 — Positively selected sites in HIV-1 viral infectivity factor (vif). stands for a positively selected site and stands for a negatively selected site (FEL ). and reflect borderline significant sites (FEL p between and ). and denote significant sites (FEL ). (PDF) [file pgen.1002764.s015.pdf]

	MEME MLE					FEL MLE		p-value		q-value	log L		
Site	α	β^-	q^-	β^+	q^+	α	β	MEME	FEL	MEME	MEME	FEL	FEL result
6	0.00	0.00	0.98	611.61	0.02	0.80	1.18	0.001	0.750	0.05	-12.97	-20.00	+
31	1.48	1.48	0.85	38.21	0.15	1.34	4.66	0.025	0.184	1.00	-43.27	-45.29	+
37	3.15	1.03	0.88	64.70	0.12	3.45	4.31	0.044	0.788	1.00	-45.21	-47.56	+
62	0.00	0.00	0.94	40.09	0.06	0.01	1.17	0.021	0.568	1.00	-19.93	-22.76	+
101	0.00	0.00	0.00	3.20	1.00	0.00	3.19	0.037	0.025	1.00	-36.93	-36.99	+ + +
109	0.00	0.00	0.82	28.07	0.18	0.00	5.17	0.000	0.007	0.05	-30.15	-33.85	+ + +
124	0.00	0.00	0.81	25.13	0.19	0.00	2.54	0.034	0.244	1.00	-33.14	-34.94	+
