# Supplementary material for: Detecting Individual Sites Subject to Episodic Diversifying Selection
Source: PLoS Genet. 2012 Jul 12;8(7):e1002764. doi: 10.1371/journal.pgen.1002764 (PMC3395634; doi:10.1371/journal.pgen.1002764)
Supplement: Table S13 — Positively selected sites in Influenza A virus hemagglutinin (H3N2 serotype). Superscript letters after the site indicate the epitope in which substitutions can affect phenotype. stands for a positively selected site and stands for a negatively selected site (FEL ). and reflect borderline significant sites (FEL p between and ). and denote significant sites (FEL ). (PDF) [file pgen.1002764.s016.pdf]

	MEME MLE					FEL MLE		p-value		q-value	log L		
Site	α	β^-	q^-	β^+	q^+	α	β	MEME	FEL	MEME	MEME	FEL	FEL result
15	2.03	0.00	1.00	529.01	0.00	2.27	0.39	0.007	0.040	0.27	-66.37	-71.02	− − −
53[C]	0.00	0.00	0.94	19.78	0.06	0.00	1.18	0.025	0.173	0.46	-45.88	-47.85	+
128	0.00	0.00	0.00	1.79	1.00	0.00	1.79	0.023	0.014	0.50	-53.74	-53.74	+ + +
133[A]	0.00	0.00	0.89	31.52	0.11	0.00	2.89	0.000	0.007	0.03	-84.92	-88.17	+ + +
135	0.48	0.00	0.86	19.19	0.14	0.57	2.62	0.009	0.071	0.30	-79.27	-81.54	++
138	0.00	0.00	0.21	5.81	0.79	0.00	4.53	0.000	0.000	0.02	-135.88	-135.84	+ + +
145[A]	0.00	0.00	0.05	4.47	0.95	0.00	4.21	0.002	0.001	0.13	-139.10	-139.09	+ + +
157	0.00	0.00	0.96	86.95	0.04	0.00	3.40	0.000	0.008	0.00	-54.13	-61.96	+ + +
159	1.95	1.40	0.98	235.09	0.02	1.95	3.33	0.000	0.375	0.02	-99.69	-107.02	+
162[E]	0.00	0.00	1.00	117.90	0.00	0.07	0.42	0.004	0.698	0.16	-15.62	-20.25	+
186	2.56	0.00	0.87	49.56	0.13	2.59	4.38	0.032	0.304	0.52	-182.30	-184.42	+
190[B]	0.00	0.00	0.28	3.15	0.72	0.00	2.26	0.024	0.016	0.49	-102.43	-102.41	+ + +
193[B]	0.64	0.64	0.00	3.96	1.00	0.64	3.95	0.029	0.019	0.50	-142.54	-142.52	+ + +
194	0.61	0.61	0.00	4.38	1.00	0.61	4.37	0.003	0.002	0.13	-96.80	-96.81	+ + +
213[D]	0.96	0.00	0.99	59.04	0.01	0.96	0.63	0.025	0.644	0.48	-33.88	-37.60	−
226	0.49	0.00	0.63	43.13	0.37	0.59	13.41	0.000	0.000	0.00	-240.52	-241.92	+ + +
275	0.00	0.00	0.00	1.62	1.00	0.00	1.62	0.022	0.014	0.51	-57.15	-57.16	+ + +
276[C]	0.00	0.00	0.00	2.39	1.00	0.00	2.39	0.010	0.006	0.31	-79.33	-79.33	+ + +
293	3.36	0.00	1.00	10000.00	0.00	3.85	0.24	0.011	0.001	0.31	-61.35	-65.66	− − −
297	0.48	0.00	1.00	114.92	0.00	0.85	0.25	0.014	0.382	0.37	-20.91	-24.81	−
