# Supplementary material for: Detecting Individual Sites Subject to Episodic Diversifying Selection
Source: PLoS Genet. 2012 Jul 12;8(7):e1002764. doi: 10.1371/journal.pgen.1002764 (PMC3395634; doi:10.1371/journal.pgen.1002764)
Supplement: Table S14 — Positively selected sites in Japanese encephalitis virus env. stands for a positively selected site and stands for a negatively selected site (FEL ). and reflect borderline significant sites (FEL p between and ). and denote significant sites (FEL ). (PDF) [file pgen.1002764.s017.pdf]

	MEME MLE					FEL MLE		p-value		q-value	$\log L$		
Site	α	β^-	q^-	β^+	q^+	α	β	MEME	FEL	MEME	MEME	FEL	FEL result
33	2.71	0.00	0.83	105.37	0.17	2.71	1.36	0.018	0.369	1.00	-54.24	-57.81	$-$
242	0.00	0.00	0.00	1.19	1.00	0.00	1.19	0.027	0.018	1.00	-22.04	-22.08	$+++$
327	1.87	0.00	0.92	1622.27	0.08	4.45	0.69	0.005	0.020	1.00	-38.71	-43.06	$---$
366	0.79	0.00	0.91	93.78	0.09	0.80	0.60	0.024	0.775	1.00	-29.59	-32.60	$-$
