# Supplementary material for: Detecting Individual Sites Subject to Episodic Diversifying Selection
Source: PLoS Genet. 2012 Jul 12;8(7):e1002764. doi: 10.1371/journal.pgen.1002764 (PMC3395634; doi:10.1371/journal.pgen.1002764)
Supplement: Table S15 — Positively selected sites in mammalian -globin. The FEL result column summarizes the classification obtained by FEL. stands for a positively selected site and stands for a negatively selected site (FEL ). and reflect borderline significant sites (FEL p between and ). and denote significant sites (FEL ). (PDF) [file pgen.1002764.s018.pdf]

	MEME MLE					FEL MLE		p-value		q-value	log L		
Site	α	β^-	q^-	β^+	q^+	α	β	MEME	FEL	MEME	MEME	FEL	FEL result
10	0.45	0.12	0.84	13.37	0.16	0.44	0.75	0.006	0.524	0.15	-40.86	-44.82	+
11	0.00	0.00	0.70	14.20	0.30	0.19	1.47	0.008	0.030	0.17	-42.45	-43.91	+ + +
14	0.81	0.00	0.82	5.57	0.18	0.81	0.83	0.042	0.974	0.50	-31.94	-34.31	+
21	0.00	0.00	0.67	2.31	0.33	0.00	0.74	0.019	0.019	0.30	-19.27	-19.66	+ + +
27	0.00	0.00	0.83	2.53	0.17	0.00	0.40	0.032	0.078	0.42	-16.42	-17.48	++
42	0.25	0.02	0.82	20.25	0.18	0.20	1.51	0.001	0.055	0.05	-38.03	-42.61	++
48	0.00	0.00	0.76	50.80	0.24	0.46	1.61	0.000	0.140	0.01	-44.76	-52.84	+
50	0.30	0.30	0.71	55.30	0.29	0.73	3.05	0.002	0.052	0.07	-52.29	-55.75	++
54	0.58	0.00	0.68	7.23	0.32	0.52	1.92	0.005	0.149	0.14	-47.02	-50.55	+
110	0.94	0.00	0.86	50.30	0.14	0.92	0.71	0.001	0.753	0.03	-34.84	-43.86	−
123	0.44	0.44	0.74	54.28	0.26	0.68	3.16	0.014	0.153	0.25	-56.36	-58.79	+
133	0.23	0.00	0.96	142.03	0.04	0.43	0.22	0.032	0.503	0.46	-18.42	-22.28	−
