# Supplementary material for: Detecting Individual Sites Subject to Episodic Diversifying Selection
Source: PLoS Genet. 2012 Jul 12;8(7):e1002764. doi: 10.1371/journal.pgen.1002764 (PMC3395634; doi:10.1371/journal.pgen.1002764)
Supplement: Table S16 — Positively selected sites in primate cytochrome c oxidase subunit 1 (COX1). stands for a positively selected site and stands for a negatively selected site (FEL ). and reflect borderline significant sites (FEL p between and ). and denote significant sites (FEL ). (PDF) [file pgen.1002764.s019.pdf]

	MEME MLE					FEL MLE		p-value		q-value	log L		
Site	α	β^-	q^-	β^+	q^+	α	β	MEME	FEL	MEME	MEME	FEL	FEL result
117	0.00	0.00	0.81	1.56	0.19	0.10	0.20	0.038	0.805	1.00	-24.96	-27.43	$+$
333	1.01	0.00	0.91	693.81	0.09	1.17	0.09	0.037	0.000	1.00	-41.81	-45.22	$---$
480	0.10	0.00	0.70	2.72	0.30	0.12	0.63	0.012	0.097	1.00	-35.80	-37.81	$++$
508	0.27	0.00	0.87	25.12	0.13	0.53	0.26	0.027	0.476	1.00	-36.93	-39.94	$-$
