# Supplementary material for: Detecting Individual Sites Subject to Episodic Diversifying Selection
Source: PLoS Genet. 2012 Jul 12;8(7):e1002764. doi: 10.1371/journal.pgen.1002764 (PMC3395634; doi:10.1371/journal.pgen.1002764)
Supplement: Table S17 — Positively selected sites in Salmonella recA. stands for a positively selected site and stands for a negatively selected site (FEL ). and reflect borderline significant sites (FEL p between and ). and denote significant sites (FEL ). (PDF) [file pgen.1002764.s020.pdf]

| | MEME MLE | | | | | FEL MLE | | p-value | | q-value | log $L$ | | |
|---|---|---|---|---|---|---|---|---|---|---|---|---|---|
| Site | $\alpha$ | $\beta^-$ | $q^-$ | $\beta^+$ | $q^+$ | $\alpha$ | $\beta$ | MEME | FEL | MEME | MEME | FEL | FEL result |
| 142 | 0.00 | 0.00 | 0.98 | 1370.53 | 0.02 | 0.00 | 0.74 | 0.046 | 0.282 | 1.00 | -12.42 | -14.16 | + |
