# Supplementary material for: Detecting Individual Sites Subject to Episodic Diversifying Selection
Source: PLoS Genet. 2012 Jul 12;8(7):e1002764. doi: 10.1371/journal.pgen.1002764 (PMC3395634; doi:10.1371/journal.pgen.1002764)
Supplement: Table S18 — Positively selected sites in vertebrate rhodopsin. stands for a positively selected site and stands for a negatively selected site (FEL ). and reflect borderline significant sites (FEL p between and ). and denote significant sites (FEL ). (PDF) [file pgen.1002764.s021.pdf]

	MEME MLE					FEL MLE		p-value		q-value	log L		
Site	α	β^-	q^-	β^+	q^+	α	β	MEME	FEL	MEME	MEME	FEL	FEL result
14	0.96	0.00	0.93	26.59	0.07	1.36	0.30	0.002	0.009	0.25	-58.28	-68.41	$---$
16	0.50	0.00	0.88	3.83	0.12	0.59	0.30	0.046	0.395	0.81	-46.78	-51.71	$-$
19	1.01	0.20	0.92	48.38	0.08	1.29	0.45	0.003	0.061	0.18	-83.46	-89.22	$--$
50	1.80	0.00	0.70	11.55	0.30	2.03	1.16	0.033	0.228	0.79	-109.11	-114.61	$-$
54	0.00	0.00	0.26	0.64	0.74	0.00	0.45	0.036	0.024	0.79	-43.22	-43.47	$+++$
84	0.00	0.00	0.93	185.13	0.07	1.07	0.09	0.004	0.006	0.20	-39.89	-44.78	$---$
93	0.37	0.00	0.97	134.05	0.03	0.51	0.17	0.005	0.145	0.18	-35.75	-42.95	$-$
96* [a]	0.54	0.00	0.98	43.40	0.02	0.93	0.11	0.009	0.047	0.29	-20.01	-27.30	$---$
144	0.00	0.00	0.86	4.36	0.14	0.00	0.41	0.000	0.127	0.12	-43.47	-49.25	$+$
165	1.18	0.00	0.76	6.43	0.24	1.05	1.05	0.038	0.989	0.75	-86.18	-90.61	$+$
183*	0.00	0.00	0.87	2.92	0.13	0.00	0.21	0.040	0.613	0.74	-36.60	-38.77	$+$
195*	0.61	0.15	0.92	12.68	0.08	0.88	0.49	0.032	0.392	0.87	-63.12	-67.86	$-$
205	0.00	0.00	0.95	109.05	0.05	0.33	0.14	0.011	0.459	0.34	-26.65	-30.54	$-$
210	2.12	0.00	0.89	27.28	0.11	2.67	0.56	0.037	0.007	0.76	-67.70	-80.11	$---$
213	0.36	0.00	0.59	12.25	0.41	0.74	1.76	0.002	0.390	0.16	-109.02	-114.02	$+$
236	0.44	0.00	0.98	58.79	0.02	0.67	0.08	0.005	0.044	0.20	-22.45	-30.26	$---$
271	0.60	0.00	0.96	7.76	0.04	0.70	0.20	0.032	0.060	0.81	-38.95	-45.02	$--$
273	0.00	0.00	0.96	9.72	0.04	0.00	0.17	0.003	0.698	0.20	-19.11	-23.99	$+$
277	0.41	0.00	0.91	14.70	0.09	0.49	0.40	0.002	0.744	0.17	-56.84	-65.88	$-$

[a] Substitutions at codons marked with * were reported to affect the wavelength of maximum absorption by Yokoyama et al. (2008)
