# Supplementary material for: Detecting Individual Sites Subject to Episodic Diversifying Selection
Source: PLoS Genet. 2012 Jul 12;8(7):e1002764. doi: 10.1371/journal.pgen.1002764 (PMC3395634; doi:10.1371/journal.pgen.1002764)
Supplement: Table S19 — Positively selected sites in West Nile virus NS3. stands for a positively selected site and stands for a negatively selected site (FEL ). and reflect borderline significant sites (FEL p between and ). and denote significant sites (FEL ). (PDF) [file pgen.1002764.s022.pdf]

	MEME MLE					FEL MLE		p-value		q-value	$\log L$		
Site	α	β^-	q^-	β^+	q^+	α	β	MEME	FEL	MEME	MEME	FEL	FEL result
249	0.00	0.00	0.00	2.50	1.00	0.00	2.55	0.010	0.005	1.00	-33.85	-34.11	$+++$
557	0.29	0.00	0.96	130.60	0.04	0.33	0.07	0.031	0.325	1.00	-13.97	-17.12	$-$
