# Supplementary material for: Detecting Individual Sites Subject to Episodic Diversifying Selection
Source: PLoS Genet. 2012 Jul 12;8(7):e1002764. doi: 10.1371/journal.pgen.1002764 (PMC3395634; doi:10.1371/journal.pgen.1002764)
Supplement: Table S20 — Test p-values for positively selected sites found by MEME in a set of vertebrate rhodopsin sequences analyzed with REL methods in Yokoyama2008fk. Sites with are shown in bold. The partial ordering of subsets is as follows: Squirrelfish Fish All, Coelacanth and tetrapods All. Sites found to be under positive selection with posterior probability of (M8 model) in Yokoyama2008fk in at least one of the subsets are marked with . (PDF) [file pgen.1002764.s023.pdf]

Codon	All ($N = 38$)	Fish ($N = 28$)	Coelacanth and tetrapods ($N = 10$)	Squirrelfish ($N = 11$)
13	0.37	1	**0.0047**	1
14	**0.0015**	**0.0035**	0.67	0.17
16	**0.046**	0.13	0.67	0.67
19	**0.0033**	**0.012**	0.67	0.13
50*	**0.033**	0.35	0.67	0.58
54	**0.036**	0.056	0.29	0.24
84	**0.0043**	0.67	**0.05**	0.67
93	**0.005**	0.67	**0.03**	1
96	**0.0086**	**0.00093**	0.67	1
136	0.23	**0.024**	0.67	1
144	**0.00038**	**0.0068**	0.076	1
165	**0.038**	0.14	0.54	0.67
183	**0.04**	0.077	1	1
195	**0.032**	0.074	**0.036**	1
198	0.1	0.064	**0.036**	0.44
205	**0.011**	**0.034**	1	0.67
210	**0.037**	0.17	0.67	0.67
213*	**0.0019**	0.15	0.14	0.54
214*	0.23	0.24	0.67	**0.025**
225	0.25	0.66	**0.036**	1
236	**0.0048**	**0.0028**	0.67	1
271	**0.032**	**0.015**	0.67	1
273	**0.003**	1	0.073	1
277	**0.0016**	**0.0045**	0.071	1
