# Supplementary material for: Detecting Individual Sites Subject to Episodic Diversifying Selection
Source: PLoS Genet. 2012 Jul 12;8(7):e1002764. doi: 10.1371/journal.pgen.1002764 (PMC3395634; doi:10.1371/journal.pgen.1002764)
Supplement: Table S21 — Test p-values for positively selected sites found by MEME in a set of influenza A virus hemagglutinin sequences (Set 3) and its various subsets, analyzed with REL methods in Chen2011fk. Sites with are shown in bold. The partial ordering of subsets is as follows: Set 4 Set 1 Set 3, Set 5 Set 2 Set 3, Set 6 Set 3, Set 7 Set 3. Sites found to be under positive selection with posterior probability of (M3 model) in Chen2011fk in at least one of the subsets are marked with . (PDF) [file pgen.1002764.s024.pdf]

Codon	Set 1	Set 2	Set 3	Set 4	Set 5	Set 6	Set 7
3*	**0.017**	0.44	0.54	**0.028**	0.67	0.35	0.67
121*	0.1	0.084	**0.039**	0.066	0.11	1	**0.041**
137*	0.056	0.076	**0.026**	0.38	1	0.34	0.38
155*	**0.0043**	**0.0048**	**0.0059**	0.55	0.4	**0.035**	0.56
159*	**0.0018**	**0.00047**	**0.0005**	**0.00083**	**0.0036**	0.4	**0.0016**
189*	0.38	0.077	**0.035**	0.32	0.34	0.34	0.33
193*	**0.013**	**0.0083**	**0.0056**	0.19	0.12	**0.0039**	0.097
226*	**0.01**	**0.0096**	**0.002**	**0.045**	**0.043**	0.23	**0.016**
