# Supplementary material for: Detecting Individual Sites Subject to Episodic Diversifying Selection
Source: PLoS Genet. 2012 Jul 12;8(7):e1002764. doi: 10.1371/journal.pgen.1002764 (PMC3395634; doi:10.1371/journal.pgen.1002764)
Supplement: Text S1 — Supplementary methods, results, and discussion. (PDF) [file pgen.1002764.s025.pdf]

Supplemental Methods

Selection of an appropriate benchmark test

Possible comparison methods against which to benchmark MEME include fixed effects (e.g. FEL in [1]) and random effects (e.g. the M series models in Yang et al [2]). We decided to choose FEL. As shown by Kosakovsky Pond and Frost [1], fixed and random effects methods have very similar statistical performance on sufficiently informative datasets where positive selection is pervasive, hence FEL is an adequate representative for all "site" methods. FEL is similar to MEME in a number of ways: neither method imposes a distribution over the synonymous and non-synonymous rates, both methods permit synonymous rate variation from site to site, and both methods use likelihood ratio tests (LRTs) at individual sites to assess significance. Consequently, any differences in performance can be attributed to the fact that MEME includes branch-to-branch variation in substitution rates at a site.

Had we chosen to compare MEME to random effects methods, the cause of the difference in performance would be more difficult to isolate. Firstly, strong distributional assumptions (required for tractable random effects methods) can influence site-specific inference. For example, the popular M2 or M8 classes of models implemented in the PAML package [3], and frequently used to detect positive selection, assume that the synonymous rate is equal for all sites in the alignment. With this assumption, it is easy to mistake relaxed selective constraints for positive selection [1]. Consequently, it would be difficult to tell whether any relative benefits of MEME were due to the freedom from distributional assumptions, to sitewise LRT inference itself, or to the feature of interest: allowing ω to vary across branches.

Accounting for recombination.

Inference of positive selection can be misled by recombination if it is not properly accounted for [4, 5]. If this phylogenetic error is large enough, it will severely mislead inference of selection. To ensure that MEME is robust to recombination, we adopt the strategy of Scheffler et al [6]: first infer recombination breakpoints [e.g. using GARD [7]] and phylogenies for the separate alignment partitions dictated by the breakpoint locations, then detect selection using a separate phylogeny for each non-recombinant fragment of the alignment.

Implementation

The MEME analysis is implemented as an option in the *QuickSelectionDetection.bf* standard analysis in the HyPhy package [8], and as a module for the Datamonkey server [`www.datamonkey.org`, [9]].

To speed up execution, the fitting of alternative and null models to each site are trivially distributed across multiple computing nodes. The estimates of α and β rates obtained from FEL are used as starting points for site-wise MEME analyses. Additionally, it is not necessary to fit the null model to those sites where the estimate of ω^+ under the alternative model does not exceed 1.

If recombination is suspected, MEME analysis should be performed on a partitioned alignment, supplied as a NEXUS file with the appropriate CHARSET blocks – for example, as output by the GARD module [10] of the Datamonkey server.

Simulated data

We evaluated the accuracy and power of MEME on sequence data simulated over a range of selective regimes.

Empirical derivation of the test statistic. As discussed by Self and Liang [11], the asymptotic distribution of the likelihood ratio test statistic when maximum likelihood estimates are at the boundaries of the parameter space can, in many cases, be described by a mixture of χ^2 distributions with different degrees of freedom. The exact form of the asymptotic distribution is very problem dependent, and in many cases is impossible to derive analytically. For example, Anisimova and Yang [12], explored the utility of a 50:50 mixture of χ^2_0 and χ^2_1 for the case of a single one-sided constraint. We surmised that under the worst-case null of strict neutrality, the asymptotic distribution of the LRT under MEME would be a mixture of χ^2_0, χ^2_1 and χ^2_2. The contribution of each of the components is exemplified by the following cases:

χ^2_0 : if the maximum likelihood estimates (MLEs) of the parameters under H_A of MEME are in the parameter subspace defined by H_0. Indeed, if $\beta^+ < \alpha$, then the likelihood ratio test is exactly 0 and effective change in the degrees of freedom is 0.

χ^2_1 : if $\alpha > 0, 0 < q^- < 1$, $\beta^- < \alpha$, and $\beta^+ > \alpha$, then the null model enforces a single constraint ($\beta^+ = \alpha$), i.e. effective change in the degrees of freedom is 1.

χ^2_2 : if $\alpha > 0, 0 < q^- < 1$, $\beta^- = \alpha$, and $\beta^+ > \alpha$, then the null model enforces two effective constraints ($\beta^+ = \beta^- = \alpha$, q^- is not identifiable), i.e. effective change in the degrees of freedom is 2.

Using the null simulations from datasets with 100 or more sequences (see next section), we generated a sample of $33,000$ LRT test values, representing a range of α - variation scenarios, tree shapes, data set sizes and divergence levels. We next numerically fitted the mixture of χ^2_0, χ^2_1 and χ^2_2 (using maximum likelihood) to the empirical distribution of the LRT statistic. All values of the LRT < 0.001 (HyPhy numerical optimization tolerance) were set to 0. We derived the asymptotic distribution of $0.33\chi^2_0 + 0.3\chi^2_1 + 0.37\chi^2_2$, which is in excellent agreement with the empirical distribution of LRT values (see Figure S1).

To further investigate the performance of the test statistic, we search Treebase.org for trees with $96-192$ leaves (expected asymptotic range for the test statistic), which included inferred branch lengths. This search returned 36 trees, with mean pairwise distances between tips ranging from 0.00874 to 0.753 substitutions per site (see Table S1). Using each tree, we generated 10 replicates under strict neutrality. Nucleotide substitution biases, alignment lengths, nucleotide base frequencies, the number and values of synonymous substitution rates were selected randomly, from the database of fit results of evolutionary fingerprinting [13] analyses (using alignments with at least 96 sequences) submitted to `datamonkey.org` over the past two years –representing a wide variety of biological data sets. The rates of false positives achieved by MEME on these alignments are summarized in Table S1.

Null simulations. We compared the performance of MEME to that of FEL, using sequence data generated under neutrality ($\omega = 1$ for every branch and every site) and variable site-to-site synonymous substitution rates, previously analyzed in Kosakovsky Pond and Frost [1]. 40 replicates with $S = 250$ codons were simulated for $N = 8, 16, 32, 64, 128$ and 256 sequences, yielding 10000 codons in total for each N.

To further evaluate the performance of MEME under the worst-case scenario null model (all branches and all sites have $\omega = 1$) on large trees, we obtained 6 phylogenies containing > 500 taxa from studies deposited in TreeBase (`http://www.treebase.org`). 10 replicates with 300 codons each were generated for each phylogeny using variable synonymous rates and $\omega = 1$: 150 codons with $\alpha = 0.52$, 100 codons with $\alpha = 0.9$, and 50 codons with $\alpha = 1.58$ (values taken from the vertebrate rhodopsin dataset, see below).

1. Tr31198 with $N = 640$ taxa, and mean pairwise nucleotide sequence divergence of 0.25. Type I error rate $= 0.049$.
2. Tr31199 with $N = 640$ taxa, and mean pairwise nucleotide sequence divergence of 0.26. Type I error rate $= 0.049$
3. Tr31201 with $N = 536$ taxa, and mean pairwise nucleotide sequence divergence of 0.33. Type I error rate $= 0.052$
4. Tr31203 with $N = 517$ taxa, and mean pairwise nucleotide sequence divergence of 0.12. Type I error rate $= 0.049$
5. Tr31210 with $N = 566$ taxa, and mean pairwise nucleotide sequence divergence of 0.18. Type I error rate $= 0.049$
6. Tr31211 with $N = 591$ taxa, and mean pairwise nucleotide sequence divergence of 0.34. Type I error rate $= 0.050$
7. Tr31214 with $N = 511$ taxa, and mean pairwise nucleotide sequence divergence of 0.33. Type I error rate $= 0.047$

Complex Null Simulations. Because the null model for MEME has a number of *a priori* unknown parameters, the distribution of the p-values, for a fixed data set and other nuisance parameters, will be a function of these parameters: $Pr\{p = P|\beta^-, \beta^+, q^-, \alpha\}$. For fixed values of the four parameters, this probability can be estimated by simulation. Previous simulations have been concerned with the case when $\beta^- = \beta^+ = \alpha, q^- = 1$ and variable α –the values on the boundary of the parameter space separating the null from the alternative, and chosen to represent the "worst case" of strict neutrality. Of course, in a biological data set one would expect the majority of sites to be under some degree of conservation, i.e. evolving under $\beta^- < \alpha$, hence the MEME is likely more conservative on such data that neutral simulations would indicate. There are a number of possible definitions of p-values for such "composite" nulls (see [14] for example); one possible definition is the predictive prior p-value, where $Pr\{p = P\}$ is integrated with respect to some (e.g. uniform) prior on $(\beta^-, \beta^+, q^-, \alpha)$. An approximate calculation of such a value is shown in Table S2 for three example trees with 62-64 tips (drawn once again from TreeBase), and a grid on β^-, β^+ values with $q^- = 0.8$. As expected, false positive rates decay rapidly as both values of β are reduced; for one of the datasets (Tr25302), false positive rates at test significance level of 0.05 range from 0 to 6.3%, with the prior predictive p of 1.9%.

Constant selection pressure at individual sites. For more biologically realistic simulations, we generated data based on the phylogenies and maximum likelihood parameter estimates derived from three empirical alignments, selected to represent different divergence levels and numbers of sequences:

1. Japanese encephalitis virus *env* [2] as an example of a small and low divergence alignment with low rates of detectable positive selection;
2. vertebrate rhodopsin [15] as an example of an intermediate size and divergence alignment with moderate rates of detectable positive selection;

3. camelid VHH [16] as an example of a large and high divergence alignment with high rates of detectable positive selection.

When simulating data under the standard Muse-Gaut 94 model, we drew values of the synonymous substitution rate α from a 3-bin distribution. The equiprobable values of α were taken as the 25%, 50% and 75% quartiles for the MLEs of site-wise α estimates for the corresponding alignment under FEL, normalized to have mean one. These distributions were as follows: $\alpha \in \{0.35, 0.9, 1.75\}$ for Japanese encephalitis virus *env*, $\alpha \in \{0.52, 0.9, 1.58\}$ for vertebrate rhodopsin, and $\alpha \in \{0.39, 0.81, 1.80\}$ for camelid VHH.

For each $\omega^+ \in \{1.25, 1.5, 1.75, 2, 3, 5, 8, 12, 16\}$, we generated 10 alignments with the same number of codons as the underlying empirical dataset, with branch lengths and nucleotide rate parameters fixed at MLE estimates under the MG94×REV model. 80% of sites were simulated as negatively selected or neutrally evolving (20% for each value $\omega = 0.25, 0.5, 0.75, 1$), evenly distributing the sites for each ω value among the three values of α. The remaining 20% of sites were simulated using the corresponding value of $\omega^+ > 1$, also evenly distributed over the three values of α. Thus, each simulated alignment contained synonymous rate variability, sites used to measure false positive rates, and sites needed to assess the power of FEL and MEME.

Variable selection pressure at individual sites. We generated sequence data where only a proportion of branches evolved with $\omega > 1$ at some sites (i.e. under the mixture model of MEME). We were particularly interested in instances where most branches were under fairly strong purifying selection, thus we selected ω^- values to be $0, 0.2$ or 0.4. We chose the expected proportion of branches under selection (q^+) to be $0.1, 0.25$ or 0.5. To measure false positive rates, we used ω^N values of $0.75, 0.9$ or 1, and we set ω^+ values to $4, 12$ or 36 to examine power. For each triplet of q^+, ω^- and ω^+ values, we simulated 10 replicates, each comprised of negative, control and detection subsets. The negative subset was obtained by simulating 20% of the sites using one of the three ω^- values, and each of the three ω^N values, mixed using $1 - q^+$ and q^+ weights, respectively. 20% of the sites were evolved neutrally (i.e. with $\omega = 1$ for all lineages); these formed the control subset. 20% of the sites were simulated with ω^+ and ω^- values mixed in the q^+ and $1 - q^+$ proportions, respectively, to form the detection subset. Thus, for each of the three baseline datasets, we produced 270 simulated alignments.

As an example, in each alignment there are 15 groups of equal (or approximately equal) numbers of sites, and within a particular group the selective regime is shared by all sites. For example, each camelid VHH simulation using $q^+ = 0.1, \omega^- = 0.4$ and $\omega^+ = 12$ has the following 15 groups of sites with one or two ω branch classes at each site:

Group	Sites	α	ω^1	$Pr\{\omega = \omega^1\}$	ω^2	$Pr\{\omega = \omega^2\}$	Designation
1	6	0.39	0.4	0.9	0.75	0.1	Negative
2	6	0.81	0.4	0.9	0.75	0.1	Negative
3	7	1.80	0.4	0.9	0.75	0.1	Negative
4	6	0.39	0.4	0.9	0.9	0.1	Negative
5	6	0.81	0.4	0.9	0.9	0.1	Negative
6	7	1.80	0.4	0.9	0.9	0.1	Negative
7	6	0.39	0.4	0.9	1.0	0.1	Negative
8	6	0.81	0.4	0.9	1.0	0.1	Negative
9	7	1.80	0.4	0.9	1.0	0.1	Negative
10	6	0.39	1	1			Control
11	6	0.81	1	1			Control
12	7	1.80	1	1			Control
13	7	0.39	0.4	0.9	12.0	0.1	Detection
14	6	0.81	0.4	0.9	12.0	0.1	Detection
15	7	1.80	0.4	0.9	12.0	0.1	Detection

Empirical Bayes inference of branches subject to episodic selection at a given site. We simulated 100 replicates using the vertebrate rhodopsin phylogeny and parameter estimates (described above), where positive selection operated along several branches specified *a priori*, and then used the empirical Bayes (EB) inference to attempt to recover these branches at individual sites. A graphical representation of the simulation scenario is shown in Figure S2. We selected both terminal and internal branches as those to evolve with $\omega = 4, 12, 36$ and varied the selection regime along the rest of the branches from strong negative, to neutral selection. Empirical Bayes inference was performed only at those sites where the MEME LRT test was significant at $p \leq 0.05$.

The results of applying EB inference to simulated data are summarized in Figure S3 and discussed in more detail in the main text.

All simulated data can be obtained in NEXUS format from `http://www.hyphy.org/pubs/MEME/simulations.tar.bz2`.

Empirical data

To gauge the comparative performance of MEME and FEL when identifying sites subject to pervasive diversifying selection, we utilized a collection of 10 alignments from [17], representing a diverse array of taxa including viruses, genes subject to differing levels of conservation, and a range of data set sizes. Four additional empirical data sets were included: the alignment of 21 NS3 gene sequences from West Nile virus isolates [18], the alignment of 38 vertebrate rhodopsin sequences [15], the alignment of 476 HIV-1 reverse transcriptase sequences sampled from a group of patients before and after exposure to

antiretroviral therapy, and the alignment of 86 influenza A virus (H3N2) hemagglutinin sequences. All empirical alignments can be downloaded from `http://www.hyphy.org/pubs/MEME/empirical.tar.bz2`.

Supplemental Results

Abalone sperm lysin This sperm-egg compatibility protein has been shown to experience both site- and lineage-specific selective pressures, using models which could only allow one or the other kind of variability [19]. Yang and Swanson [20] hypothesized and showed that solvent exposed sites evolve with higher mean ω than the buried residues. In a MEME analysis (Table S4), 23 out of 88 exposed residues are identified as positively selected, while only 3 of 46 buried residues are reported as such ($p = 0.02$, Fisher's exact test). As with β-globin, MEME to resolve borderline FEL cases, but also identifies 2 sites which are assessed as negatively selected by FEL.

Diatom silicon transporters (SIT) Silicon uptake is an important component in diatom cell wall construction, but physiological differences exist in the amount and efficiency of silica use between freshwater and marine diatoms. Previous analysis [21] of a silicon transporter gene found no evidence for positive selection in freshwater and marine diatoms, and FEL analysis corroborated this finding; unexpectedly, MEME found evidence of positive selection at 48 sites in this silicon transporter gene, and all but two of these sites were determined by FEL to be under some degree of purifying selection (Table S6). Notably, we found no significant association between the number of positively selected sites and membership to transmembrane versus intermembrane regions: 19/142 versus 29/157 sites respectively (Chi-square test, $\alpha = 0.05$). Freshwater diatoms are polyphyletic and adaptation in the silicon transporter gene would likely occur only in part of the phylogeny. Therefore, it is not unexpected that previous methods failed to detect sites under positive selection, as it was likely to be episodic along the diatom phylogeny.

Drosophila *adh*. The drosophila *adh* is a canonical example of a non-neutrally evolving locus [22], possibly due to balancing selection [23]. Yang et al [2] could not find evidence of positive selection in this alignment using lineage-homogeneous models, and concluded that the result "... may be due to the lack of power of our models to detect balancing selection". MEME detects 14 sites subject to strong (mean $\beta^+ = 65$) but episodic (mean $q^+ = 0.08$) selection – a pattern not inconsistent with the action of balancing selection (Table S7).

Echinoderm H3 Histones, a major component of chromatin, are involved in packing DNA are among the most conserved proteins in the Eukaryotic domain. Their evolution is dominated by strong and pervasive purifying selection [24], and FEL and MEME confirm this pattern in the echinoderm H3 family. Surprisingly, MEME identified a single codon, site 5, as being under episodic positive selection, even though FEL found this site to be under strong purifying selection (Table S8). However, a closer look at this site reveals a more nuanced evolutionary history. Every extant echinoderm taxon included here encodes a serine at site 5; however, different groups of echinoderms encode the 4-fold redundant version, whereas others encode the 2-fold redundant version. There appear to have been at least two shifts between these codons, as members of families Asteroidea and Ophiuroidea encode both versions of the serine codon. Due to a lack of sequence information for basal lineages, we were unable to determine the ancestral version of this serine codon. Nevertheless, under the model assumption that multiple changes at the same site do not occur simultaneously, positive selection appears to have acted repeatedly at this site to maintain primary protein structure. Unlike previous methods, MEME is able to capture this selective phenomenon.

Flavivirus NS5. Tick-borne flaviviruses are a diverse and divergent group of RNA viruses, capable of infecting a wide array of species. Previous work by Yang et al [2] suggested that strong purifying selection predominates across the NS5 gene region, one of the most conserved parts of the flavivirus polyprotein. This result was not surprising given the vast phylogenetic distance separating the viruses represented in this dataset. Our analysis of a portion of the NS5 gene illustrates the capabilities of MEME relative to FEL. Specifically, MEME suggests that one site (46), found by FEL to be under strong purifying selection, were subjected to episodic positive selection (Table S9). Additionally, MEME detected two sites under positive selection, which FEL found to be under weak purifying selection (43, 175).

Hepatitis D virus Ag. Within the small antigenic protein, many of the sites identified by FEL as being under positive selection were also found by [25] using a REL method: 24, 122, 140, and 150. MEME inferred an additional 16 sites under positive selection, including four sites that FEL identified as being under purifying selection (Table S10). These sites were spread out across the functional domains of the protein, including the RNA binding region, the nuclear localization signal, and B- and T-cell epitopes [25]. Notably, our findings extend an observation by Anisimova and Yang [25] that the hydrophobic residues in a heptad repeat region are exceptionally likely to be under strong positive selection (e.g. sites 24, 28, 31 and 35 were identified by MEME). This pattern of selection is missed by the FEL analysis.

HIV reverse transcriptase (*rt*). This dataset consists of pairs of sequences from patients undergoing an antiretroviral therapy regimen containing agents that inhibit reverse transcription. One sequence from the pair was obtained before treatment, and the other following its initiation. Consequently, one would expect that only some of the lineages in this tree – those where the virus was exposed to antiretrovirals but insufficiently suppressed to develop resistance (e.g. due to pre-existing drug resistance mutations or lack of adherence) – would be subject to selection. MEME is able to identify 10 sites (Table S11) which have a well-characterized impact on the drug-resistance phenotype [26], while FEL identifies only one such site (103), and reports that three other sites (151,181, and 190) are under negative selection, on average.

HIV-1 viral infectivity factor (*vif*). HIV-1 *vif* is primarily responsible for counteracting the APOBEC3G anti-viral factor in host cells [27], and is present in all known primate lentiviruses [28]. Since we reused a sample of recent HIV-1 subtype B sequences [2], which are unlikely to have experienced species-level adaptation events, the primary selective force acting upon these sequences is immune selection (e.g. exerted by cytotoxic T-Lymphocytes). MEME appears to be boosting the power of FEL for this dataset (Table S12).

Influenza A virus H3N2 HA. Hemagglutinin is one of the two antigenic proteins of the influenza A virus; five immunologically important epitopes have been well-characterized within this protein. MEME identifies sites under positive selection in each of these five epitopes (see [29] and Table S13). Nine of the 26 sites MEME inferred to be under positive selection fall within these five epitopes. Three of them (53, 213, and 162) were missed by FEL. In addition, MEME found several positively selected sites that are thought to be involved in host adaptation: 190, 193, 194, 226, and 228. For example, substitutions at site 190 are known to affect the sialic acid receptor sensitivity switch involved in avian to human cross-species transmission. Sites 226 and 228 are also known to affect host adaptation [30, 31]. Note that two influenza A virus datasets were analyzed. We focus on the analysis of only the larger dataset; the second dataset is smaller, and is included to better understand the effect of sampling on the performance of our selection detection method.

Mammalian β-globin. This is a protein that appears to be subject to extensive selection, both based on lineage-constant models of Yang et al [2] and mutation-selection models [32]. Most sites identified by MEME are also inferred to have $\omega > 1$ on average by FEL, but the sample or effect sizes are too small to reach significance (Table S15).

Primate COXI. Episodic evolution of nuclear encoded subunits of cytochrome oxidase subunits in primate lineages has been well documented (e.g. along the lineages ancestral to anthropoid primates and hominids). Mitochondrial subunit I is involved in a number of structural interactions with other subunits, and has been hypothesized to co-evolve with them [33]. For example, site 480, detected to be under episodic positive selection (Table S16), is in direct contact with adaptively evolving subunit VIII [34]. Sites 117 and 333 are on the boundaries of structurally important $\alpha-$helices III and IX [35] respectively, and have undergone substitutions in the anthropoid primates clade.

Salmonella *recA* The *Salmonella* RecA protein is involved with essential cellular functions such as DNA repair and homologous recombination, making it a likely target of strong and uniform purifying selection [36]. FEL did not detect the presence of any sites under significant positive selection (Table S17); MEME largely confirmed this finding, though site 142 was determined to be under episodic positive selection. This site represents an instance of a single non-synonymous change at a codon where no synonymous changes were observed. Overall, the MEME analysis suggests that purifying selection pervades in *recA* evolution in *Salmonella*.

West Nile virus NS3. West Nile virus NS3 protein contains a site, 249, which is known to control virulence in American crows and was previously found to be under positive selection [18]; both MEME and FEL analysis indicated the action of strong positive selection at this site (Table S19). Two other sites were also found by MEME to be under positive selection, while FEL found weak evidence for negative selection at these sites.

Recombination

We re-analyzed two datasets while accounting for recombination: Hepatitis D virus Ag, and Japanese encephalitis virus *env*. Similar to our findings for FEL [7], a partitioning analysis alters the list of sites inferred to be under selection, but generally not in a dramatic fashion. Nonetheless, we recommend that unless recombination can be ruled unlikely based on biological considerations (e.g. as it can be for Influenza A virus intra-segment analysis, mitochondrial DNA, or HIV-1 sequences prescreened for evidence of recombination), it is prudent to perform a partitioned analysis by default.

The effect of sequence sampling.

In the Chen et al [37] study, a REL analysis of 86 IAV H3N2 hemagglutinin sequences (Set 3 in the reference) found 6 sites with empirical posterior probability of $> 95\%$ of positive selection (M3 model in Table 1 of the cited manuscript). The

analysis of a subset of 43 of those sequences (Set 2) found many more (18) sites under selection, while the analysis of the other 43 sequences (Set 1) found none. This level of variability is nontrivial because it fundamentally alters the conclusion about the extent of positive selection on a gene. One possible explanation for this finding is that selection on the entire data set has acted in an episodic fashion, but these episodes may be swamped out by predominantly purifying selection in the rest of the tree depending on the total amount of evolutionary time spent in either selective regime – something that can be strongly affected by sampling. A MEME analysis of the same sequences identified 7 sites under selection in Set 3, 4 in Set 2 (4/4 shared with Set 3) and 5 in Set 1 (4/5 shared with Set 3), see Table S21. These results, while still variable between data sets, are considerably more stable.

Hepatitis D virus Ag GARD found evidence for a breakpoint at nucleotide 254. This site is located between known functional protein domains [25], which supports the inference that it is a true recombination breakpoint. Running MEME on the trees inferred by GARD identified a list of sites that overlaps substantially with the list from the single-phylogeny analysis. In both analyses, 24 sites were identified as being under diversifying selection, and 21 of these were common to both analyses.

Japanese encephalitis virus *env* In this dataset, GARD identified four breakpoints at nucleotides 258, 624, 768, and 1092. After accounting for recombination, 3 sites were detected as being under selection (cf 4 if recombination is not corrected for). Sites 33 and 242 are common to both analyses. Site 132 ($p = 0.03$) is only detected if recombination is accounted for, but sites 327 ($p = 1.00$) and 366 ($p = 0.10$) are no longer significant.

Supplementary Discussion

Comparison with covarion models

Compound Markov models in the covarion framework [38] have also been applied to allow selective modes to switch over time. For example, Guindon et al [39] describe a model where switches occur between three different processes, each with their own ω. While the covarion model is more general than MEME in some respects (see below), its computational complexity is non-linear in the number of rate classes (MEME is linear). Because the rates at which the covarion process switches between ω classes are shared by all sites, it may be difficult to interpret evidence of selection at a single site because of smoothing effects. Indeed, Guindon et al [39] outlined how the covarion model found fewer sites under selection than traditional site models, because they sought to "detect sites in the alignment where positive selection is likely to have occurred in most of the lineages", which is exactly the setting where simpler site models perform adequately. Contrary to their approach, we designed MEME specifically to boost the power to detect sites in the alignment where positive selection is likely to have occurred only in a small (but nonempty) subset of lineages.

Sensitivity to alignment size

Two recent reports [15, 37] describe instances when the analysis of an alignment for positively selected sites using a sitewise random effects (REL) method produced a list of sites smaller than the list obtained by an analysis of a subset of the entire alignment. Puzzlingly, Yokoyama et al [15], attributed this behavior to excessive false positive rates of REL methods when applied to closely related sequences – a speculative statement convincingly refuted by a number of other studies [e.g. [40, 41]]. Chen et al [37] drew a different conclusion: "Low sensitivity of the analysis may result from that some sites under positive selection in the gene are also under negative (purifying) selection simultaneously for functional constrains, and so their ω ratios could be < 1".

We strongly agree with this sentiment; we explicitly developed MEME to reliably detect such transient selective episodes. Unlike cited constant ω analyses, MEME results are considerably more stable on these two examples, when different alignment subsets are analyzed. More sites are generally found under episodic selection in larger data sets, although p-values for selection at individual sites may be lower in small alignments: MEME does not eliminate sampling variance, but it does reduce it.

References

1. Kosakovsky Pond SL, Frost SDW (2005) Not so different after all: a comparison of methods for detecting amino acid sites under selection. Mol Biol Evol 22: 1208–1222.

2. Yang ZH, Nielsen R, Goldman N, Pedersen AMK (2000) Codon-Substitution Models for Heterogeneous Selection Pressure at Amino Acid Sites. Genetics 155: 431–449.

3. Wong WSW, Yang Z, Goldman N, Nielsen R (2004) Accuracy and power of statistical methods for detecting adaptive evolution in protein coding sequences and for identifying positively selected sites. Genetics 168: 1041-51.

4. Anisimova M, Nielsen R, Yang Z (2003) Effect of Recombination on the Accuracy of the Likelihood Method for Detecting Positive Selection at Amino Acid Sites. Genetics 164: 1229-1236.

5. Shriner D, Nickle DC, Jensen MA, Mullins J (2003) Potential impact of recombination on sitewise approaches for detecting positive natural selection. Genet Res 81: 115-121.

6. Scheffler K, Martin DP, Seoighe C (2006) Robust inference of positive selection from recombining coding sequences. Bioinformatics 22: 2493-9.

7. Kosakovsky Pond SL, Posada D, Gravenor MB, Woelk CH, Frost SDW (2006) Automated phylogenetic detection of recombination using a genetic algorithm. Mol Biol Evol 23: 1891-901.

8. Kosakovsky Pond SL, Frost SDW, Muse SV (2005) HyPhy: hypothesis testing using phylogenies. Bioinformatics 21: 676-9.

9. Delport W, Poon AFY, Frost SDW, Kosakovsky Pond SL (2010) Datamonkey 2010: a suite of phylogenetic analysis tools for evolutionary biology. Bioinformatics 26: 2455-7.

10. Kosakovsky Pond SL, Posada D, Gravenor MB, Woelk CH, Frost SDW (2006) GARD: a genetic algorithm for recombination detection. Bioinformatics 22: 3096-8.

11. Self SG, Liang KY (1987) Asymptotic Properties of Maximum Likelihood Estimators and Likelihood Ratio Tests Under Nonstandard Conditions. J Am Stat Assoc 82: 605-310.

12. Anisimova M, Yang Z (2007) Multiple hypothesis testing to detect lineages under positive selection that affects only a few sites. Mol Biol Evol 24: 1219-28.

13. Pond SLK, Scheffler K, Gravenor MB, Poon AFY, Frost SDW (2010) Evolutionary fingerprinting of genes. Mol Biol Evol 27: 520-36.

14. Bayarri M, Berger J (2000) P values for composite null models. Journal of the American Statistical Association 95: 1127–1142.

15. Yokoyama S, Tada T, Zhang H, Britt L (2008) Elucidation of phenotypic adaptations: Molecular analyses of dim-light vision proteins in vertebrates. Proc Natl Acad Sci U S A 105: 13480-5.

16. Harmsen M, Ruuls R, Nijman I, Niewold T, Frenken L, et al. (2000) Llama heavy-chain V regions consist of at least four distinct subfamilies revealing novel sequence features. Molecular Immunology 37: 579-590.

17. Kosakovsky Pond SL, Muse SV (2005) Site-to-Site Variation of Synonymous Substitution Rates. Mol Biol Evol 22: 2375-2385.

18. Brault AC, Huang CYH, Langevin SA, Kinney RM, Bowen RA, et al. (2007) A single positively selected West Nile viral mutation confers increased virogenesis in American crows. Nat Genet 39: 1162-6.

19. Yang Z, Swanson WJ, Vacquier VD (2000) Maximum-likelihood analysis of molecular adaptation in abalone sperm lysin reveals variable selective pressures among lineages and sites. Mol Biol Evol 17: 1446-55.

20. Yang Z, Swanson W (2002) Codon-Substitution Models to Detect Adaptive Evolution that Account for Heterogeneous Selective Pressures Among Site Classes. Molecular Biology And Evolution 19: 49-57.

21. Alverson A (2007) Strong purifying selection in the silicon transporters of marine and freshwater diatoms. Limnology and oceanography : 1420–1429.

22. McDonald JH, Kreitman M (1991) Adaptive protein evolution at the Adh locus in Drosophila. Nature 351: 652-4.

23. Kreitman M, Hudson RR (1991) Inferring the evolutionary histories of the Adh and Adh-dup loci in Drosophila melanogaster from patterns of polymorphism and divergence. Genetics 127: 565-82.

24. Janies D, Voight J, Daly M (2011) Echinoderm phylogeny including Xyloplax, a progenetic asteroid. Systematic biology 60: 420–438.

25. Anisimova M, Yang Z (2004) Molecular Evolution of the Hepatitis Delta Virus Antigen Gene: Recombination or Positive Selection? Journal of Molecular Evolution 59: 815-826.

26. Johnson VA, Brun-Vézinet F, Clotet B, Günthard HF, Kuritzkes DR, et al. (2010) Update of the drug resistance mutations in HIV-1: December 2010. Top HIV Med 18: 156-63.

27. Sheehy AM, Gaddis NC, Choi JD, Malim MH (2002) Isolation of a human gene that inhibits HIV-1 infection and is suppressed by the viral Vif protein. Nature 418: 646-50.

28. Gifford RJ, Katzourakis A, Tristem M, Pybus OG, Winters M, et al. (2008) A transitional endogenous lentivirus from the genome of a basal primate and implications for lentivirus evolution. Proc Natl Acad Sci U S A 105: 20362-7.

29. Wiley DC, Wilson IA, Skehel JJ (1981) Structural identification of the antibody-binding sites of Hong Kong influenza haemagglutinin and their involvement in antigenic variation. Nature 289: 373-8.

30. Rogers GN, Paulson JC, Daniels RS, Skehel JJ, Wilson IA, et al. (1983) Single amino acid substitutions in influenza haemagglutinin change receptor binding specificity. Nature 304: 76-8.

31. Connor RJ, Kawaoka Y, Webster RG, Paulson JC (1994) Receptor specificity in human, avian, and equine H2 and H3 influenza virus isolates. Virology 205: 17-23.

32. Rodrigue N, Philippe H, Lartillot N (2010) Mutation-selection models of coding sequence evolution with site-heterogeneous amino acid fitness profiles. Proc Natl Acad Sci U S A 107: 4629-34.

33. Wu W, Schmidt TR, Goodman M, Grossman LI (2000) Molecular evolution of cytochrome c oxidase subunit I in primates: is there coevolution between mitochondrial and nuclear genomes? Mol Phylogenet Evol 17: 294-304.

34. Goldberg A, Wildman DE, Schmidt TR, Huttemann M, Goodman M, et al. (2003) Adaptive evolution of cytochrome c oxidase subunit VIII in anthropoid primates. Proc Natl Acad Sci U S A 100: 5873-8.

35. Tsukihara T, Aoyama H, Yamashita E, Tomizaki T, Yamaguchi H, et al. (1996) The whole structure of the 13-subunit oxidized cytochrome c oxidase at 2.8 $\AA$. Science 272: 1136-44.

36. McQuiston J, Herrera-Leon S, Wertheim B, Doyle J, Fields P, et al. (2008) Molecular phylogeny of the salmonellae: relationships among Salmonella species and subspecies determined from four housekeeping genes and evidence of lateral gene transfer events. Journal of bacteriology 190: 7060–7067.

37. Chen J, Sun Y (2011) Variation in the analysis of positively selected sites using nonsynonymous/synonymous rate ratios: an example using influenza virus. PLoS One 6: e19996.

38. Tuffley C, Steel M (1998) Modeling the covarion hypothesis of nucleotide substitution. Mathematical biosciences 147: 63–91.

39. Guindon S, Rodrigo AG, Dyer KA, Huelsenbeck JP (2004) Modeling the site-specific variation of selection patterns along lineages. Proceedings of the National Academy of Sciences of the United States of America 101: 12957–12962.

40. Anisimova M, Bielawski JP, Yang Z (2002) Accuracy and power of bayes prediction of amino acid sites under positive selection. Mol Biol Evol 19: 950-8.

41. Yang Z, dos Reis M (2011) Statistical properties of the branch-site test of positive selection. Mol Biol Evol 28: 1217-28.
